# Exploring the Potential of Electroencephalography Signal–Based Image Generation Using Diffusion Models: Integrative Framework Combining Mixed Methods and Multimodal Analysis

**DOI:** 10.2196/72027

**Published:** 2025-06-25

**Authors:** Chi-Sheng Chen, Shao-Hsuan Chang, Che-Wei Liu, Tung-Ming Pan

**Affiliations:** 1 Department of Biomedical Engineering Chang Gung University Taoyuan Taiwan; 2 Department of Ophthalmology Linkou Chang Gung Memorial Hospital Taoyuan Taiwan; 3 School of Medicine National Tsing Hua University Taipei Taiwan; 4 Department of Orthopedics Cathay General Hospital Taipei Taiwan; 5 Department of Electronics Engineering Chang Gung University Taoyuan Taiwan; 6 Division of Urology Chang Gung Memorial Hospital Linkou Medical Center Taoyuan Taiwan

**Keywords:** electroencephalography, brain-computer interface, diffusion models, multimodal generative framework, electroencephalography to image

## Abstract

**Background:**

Electroencephalography (EEG) has been widely used to measure brain activity, but its potential to generate accurate images from neural signals remains a challenge. Most EEG-decoding research has focused on tasks such as motor imagery, emotion recognition, and brain wave classification, which involve EEG signal analysis and classification. Some studies have explored the correlation between EEG and images, primarily focusing on EEG-image pair classification or transformation. However, EEG-based image generation remains underexplored.

**Objective:**

The primary goal of this study was to extend EEG-based classification to image generation, addressing the limitations of previous methods and unlocking the full potential of EEG for image synthesis. To achieve more meaningful EEG-to-image generation, we developed a novel framework, Neural-Cognitive Multimodal EEG-Informed Image (NECOMIMI), which was specifically designed to generate images directly from EEG signals.

**Methods:**

We developed a 2-stage NECOMIMI method, which integrated the novel Neural Encoding Representation Vectorizer (NERV) EEG encoder that we designed with a diffusion-based generative model. The Category-Based Assessment Table (CAT) score was introduced to evaluate the semantic quality of EEG-generated images. In addition, the ThingsEEG dataset was used to validate and benchmark the CAT score, providing a standardized measure for assessing EEG-to-image generation performance.

**Results:**

The NERV EEG encoder achieved state-of-the-art performance in several zero-shot classification tasks, with an average accuracy of 94.8% (SD 1.7%) in the 2-way task and 86.8% (SD 3.4%) in the 4-way task, outperforming models such as Natural Image Contrast EEG, Multimodal Similarity-Keeping Contrastive Learning, and Adaptive Thinking Mapper ShallowNet. This highlighted its superiority as a feature extraction tool for EEG signals. In a 1-stage image generation framework, EEG embeddings often resulted in abstract or generalized images such as landscapes instead of specific objects. Our proposed 2-stage NECOMIMI architecture effectively extracted semantic information from noisy EEG signals, showing its ability to capture and represent underlying concepts derived from brain wave activity. We further conducted a perturbation study to test whether the model overly depended on visual cortex EEG signals for scene-based image generation. The perturbation of visual cortex EEG channels led to a notable increase in Fréchet inception distance scores, suggesting that our model relied heavily on posterior brain signals to generate semantically coherent images.

**Conclusions:**

NECOMIMI demonstrated the potential of EEG-to-image generation, revealing the challenges of translating noisy EEG data into accurate visual representations. The novel NERV EEG encoder for multimodal contrastive learning reached state-of-the-art performance both on *n*-way zero-shot and EEG-informed image generation. The introduction of the CAT score provided a new evaluation metric, paving the way for future research to refine generative models. In addition, this study highlighted the significant clinical potential of EEG-to-image generation, particularly in enhancing brain-machine interface systems and improving quality of life for individuals with motor impairments.

## Introduction

### Background

Electroencephalography (EEG) is one of the ancient techniques for measuring neuronal activity in the human brain [1,2]. EEG records electrical activity from the scalp, offering exceptional temporal resolution and high sensitivity to neural processes. Its application has significant value in clinical practice, especially for diagnosing conditions such as epilepsy, depression, and sleep disorders [3-5], as well as assessing dysfunctions in sensory transmission pathways [6,7]. Historically, the analysis of EEG signals was limited to visual inspection of changes in amplitude and frequency over time. However, with the advent of digital technology, the methodology has undergone significant evolution, enabling a more comprehensive analysis of both the temporal and spatial characteristics of these signals [8]. As a result of these advancements, EEG has become recognized as a powerful tool for capturing real-time brain activity, particularly in the subsecond range. Despite these advantages, EEG traditionally suffers from low spatial resolution, which makes it difficult to precisely identify the brain areas responsible for the observed neuronal activity at the scalp [9]. In recent years, there has been growing interest in leveraging EEG for more advanced applications such as image recognition and reconstruction [10]. These developments have led to significant improvements in the accuracy of image recognition tasks, highlighting EEG’s potential as a bridge between neural activity and visual representation [11,12]. The appeal of using EEG for image recognition lies in its ability to capture the temporal dynamics of neuronal activity, although its spatial resolution remains a major challenge. To address this, innovative methodologies, including deep learning techniques and generative models such as generative adversarial networks (GANs) [13] and diffusion models [14], have enhanced the accuracy and effectiveness of EEG-based systems, allowing for the generation of photorealistic images from neural signals [12,15,16].

Recent studies have demonstrated the feasibility of decoding natural images from EEG signals by aligning EEG responses with paired image stimuli [17]. However, most of the existing works claiming to generate images from EEG signals are still primarily image-to-image transformations. In these approaches, EEG information is typically used to slightly alter the input image, often by adding noise rather than generating new images directly from the brain signals [17-19]. In a typical experiment designed to study brain responses related to visual processes, participants view a series of images while their brain signals are recorded by a brain scanner or other recording device for subsequent analysis. Various noninvasive methods, such as functional magnetic resonance imaging (fMRI), EEG, and magnetoencephalography, can be used to capture these brain responses, each offering different sensitivity levels. Despite the availability of these techniques, there is still limited understanding regarding the precise interpretation of the data and, more importantly, the mechanisms underlying these responses. A pioneering study explored the possibility of generating impressions of what participants saw by using fMRI data based on a large image dataset sourced from YouTube [20]. However, this method faces significant challenges, including the complexity and high costs associated with using fMRI scanners. To overcome these limitations, much of the recent research has shifted toward using electrophysiological responses, particularly EEG. Although EEG offers a lower spatial resolution than fMRI, it provides much higher temporal resolution, making it a more accessible and cost-effective method. This shift to EEG is further supported by its ability to capture rapid, real-time neural dynamics. However, EEG data present their own set of challenges. The inherent noise and susceptibility to external factors complicate the accurate reconstruction of the original visual stimulus, limiting its effectiveness in image recognition and generation tasks. As a result, fMRI remains the preferred method in many studies focusing on image recognition or generation from brain signals [21], whereas EEG is used less frequently due to its noisier nature. Previous efforts such as Brain2Image and EEG-GAN have explored EEG-guided image reconstruction using variational autoencoders (VAEs) and GAN frameworks, respectively. However, most of these approaches relied heavily on paired image inputs and did not fully explore direct EEG-to-image synthesis.

To address these limitations and achieve more meaningful EEG-to-image generation, we introduced Neural-Cognitive Multimodal EEG-Informed Image (NECOMIMI), an innovative framework that focuses on EEG-based image generation. This framework integrates advanced diffusion model techniques to enhance the accuracy and realism of images generated from EEG signals. In this study, we first proposed a novel EEG encoder, Neural Encoding Representation Vectorizer (NERV), which achieved state-of-the-art (SOTA) performance in multimodal contrastive learning tasks.

### Related Work

The concept of using EEG signals for image-related tasks has evolved over time. Initially, efforts focused on decoding visual categories from EEG signals [11], demonstrating the feasibility of EEG-based image classification using deep learning models. While these early studies laid the groundwork, the datasets used were small, limiting the generalizability of the findings. However, subsequent advancements in generative models, such as VAEs and GANs, paved the way for more sophisticated EEG-to-image generation. The VAE, as proposed by Kingma and Welling [22,23], provided a means of generating and reconstructing data by learning latent data distributions. Similarly, the GAN framework introduced by Goodfellow et al [13] leveraged adversarial training between a generator and a discriminator to produce realistic images. Building on these ideas, Brain2Image [12] became the first to apply VAEs to guide image generation from EEG features. Later, EEG-GAN [18] introduced the first EEG-based image generation model using long short-term memory [24] to extract EEG features and guide the GAN’s image generation process.

Several works based on GANs, such as ThoughtViz [25], visual-guided GAN with visual-consistent term [26], BrainMedia [27], and EEG2IMAGE [16], have emerged, each focusing on improving the interaction between the EEG encoder and the GAN architecture. Despite these advancements, a common challenge remains in effectively using EEG data to guide image generation and reconstruction. This challenge persisted until the advent of Contrastive Language-Image Pretraining (CLIP; OpenAI) [28], which provided a better solution by enabling more reliable alignment between EEG and image data. EEG-CLIP [29] was the first framework to use contrastive learning to align EEG and image data. However, it was largely exploratory, and its framework was not extended for downstream tasks such as zero-shot image recognition. The next step in this evolution was to design more effective EEG encoders for contrastive learning, leveraging the rich image embeddings extracted from CLIP-based pretrained models. Recent efforts such as Natural Image Contrast EEG (NICE), Multimodal Similarity-Keeping Contrastive Learning (MUSE), Adaptive Thinking Mapper, and others [30-33] have explored this direction. In addition, some researchers have ventured into quantum-classical hybrid computing using a quantum EEG encoder [34], aiming to perform quantum contrastive learning [35]. Currently, many studies focus on zero-shot classification, where the model is tested on unseen EEG data and images, aiming to assess its ability to generalize to novel data. This is a challenging task that involves computing similarity scores between EEG and image data for recognition purposes. The evolution of contrastive learning techniques for EEG-based image recognition has improved the generalization performance of models, particularly when tested on out-of-sample data.

Despite the significant progress, the use of diffusion models to generate EEG-based images remains a relatively new area. While there are several EEG-based image reconstruction models that use diffusion models, such as NeuroVision [19], DreamDiffusion [17], diffusion model for the reconstruction from EEG to image [36], BrainViz [37], NeuroImagen [38], and EEGVision [39], most of these approaches rely on image-based features and treat EEG data as supplementary information. However, these methods primarily use images as input and are not designed to process nonvisual signals such as EEG directly. To address this gap, we proposed NECOMIMI, a flexible and modular framework explicitly designed for EEG-to-image generation. Unlike previous methods that focus on recognition or classification, NECOMIMI leverages modern diffusion models using EEG signals as the primary prompt, enabling direct generation of images from neural features. This shift represents a crucial step forward, reframing EEG not merely as a support signal but as a core semantic driver for brain-conditioned multimodal generation.

On the basis of the trajectory of prior work, it becomes clear that the field is moving from EEG-assisted image recognition toward more ambitious goals such as direct image generation conditioned solely on EEG signals. Although recent studies have explored advanced paradigms, including contrastive learning frameworks and hybrid quantum models, most still relegate EEG to a secondary role. In contrast, our preliminary investigations into quantum-enhanced EEG processing, such as quantum machine learning for enhanced EEG encoding and quantum contrastive learning [35], demonstrate how EEG can serve as the central signal in generative tasks. Quantum machine learning for enhanced EEG encoding [34] uses variational quantum circuits to enrich EEG representations with greater expressive capacity, whereas quantum contrastive learning integrates quantum projections into contrastive learning pipelines to improve cross-modal alignment. These findings underscore a critical limitation in current literature: the lack of a dedicated generative framework that places EEG at the forefront of image synthesis. Our work aimed to fill this void by shifting the paradigm from recognition to generation, positioning EEG not as an auxiliary cue but as a rich, semantic prompt capable of guiding diffusion-based image creation. This reorientation opens new avenues for decoding cognitive states through generative modeling and facilitates the development of brain-native, multimodal artificial intelligence systems.

In this study, we developed a comprehensive 2-stage EEG-to-image multimodal generative framework, which extended previous contrastive learning between EEG and images and applied it to image generation. Unlike previous works that have focused primarily on image-to-image generation with EEG features serving as guidance, our framework offered a more holistic approach to leveraging EEG data for image creation. While EEG data are traditionally characterized by high temporal but low spatial resolution, our goal was not to recover precise pixel-level representations. Instead, we treated EEG as a high-level cognitive modulator that captures semantic concepts, which can be used to guide image generation via latent diffusion models. To address the conceptual differences between EEG-to-image and traditional text-to-image tasks, we introduced a new evaluation metric, the Category-Based Assessment Table (CAT) score. This score assessed image generation performance based on semantic concepts as opposed to merely evaluating image distribution. We further established a CAT score benchmark using a vision language model on the ThingsEEG dataset. Finally, our study uncovered several notable findings and phenomena that shed light on the EEG-to-image generation process.

## Methods

### Datasets and Preprocessing

**Figure 1:**
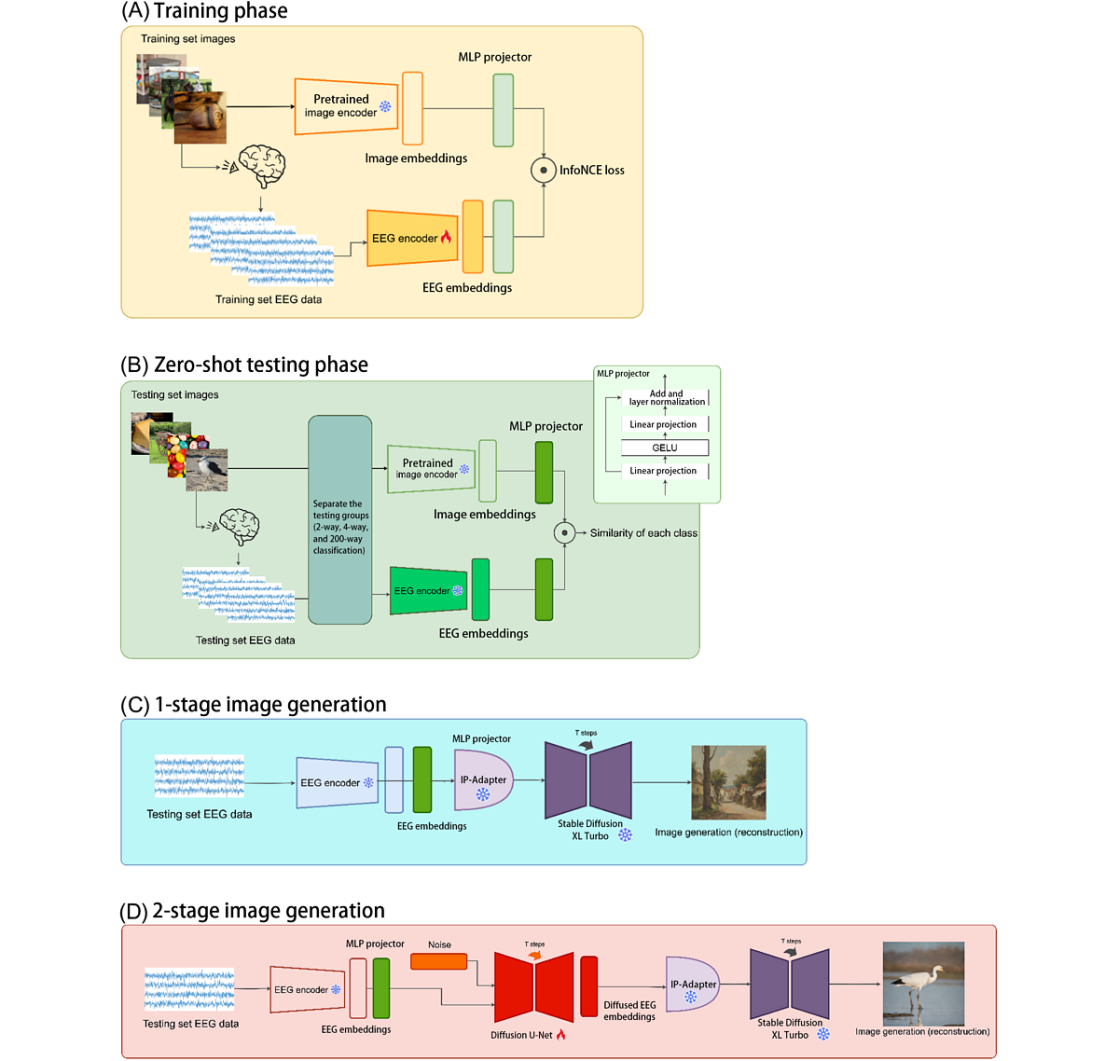
The entire workflow of the electroencephalography (EEG)–based image generation model, including (A) training phase, (B) zero-shot testing phase, (C) 1-stage image generation, and (D) 2-stage image generation. GELU: Gaussian error linear unit; InfoNCE: information noise-contrastive estimation; MLP: multilayer perceptron; SDXL: Stable Diffusion XL.

The ThingsEEG dataset comprised a large set of EEG recordings, which were obtained using a rapid serial visual presentation paradigm [40]. The rapid serial visual presentation is a commonly used experimental protocol where participants are shown a series of visual stimuli in quick succession, typically at rates of ≥10 Hz, to evoke reliable neural responses. Responses were collected from 10 participants, each of whom viewed a total of 16,740 natural images sourced from the THINGS database [41]. The dataset includes 1654 training categories, with each category containing 10 images, as well as 200 test categories, each featuring a single image. The EEG data were recorded using 64-channel Easycap equipment, and preprocessing involved segmenting the data into trials that spanned 0 to 1000 milliseconds after the stimulus was shown, with baseline correction applied based on the prestimulus period. EEG responses for each image were averaged over multiple repetitions. The workflow and models used in the EEG-based image generation system are shown in Figure 1.

### NERV EEG Encoder

**Figure 2:**
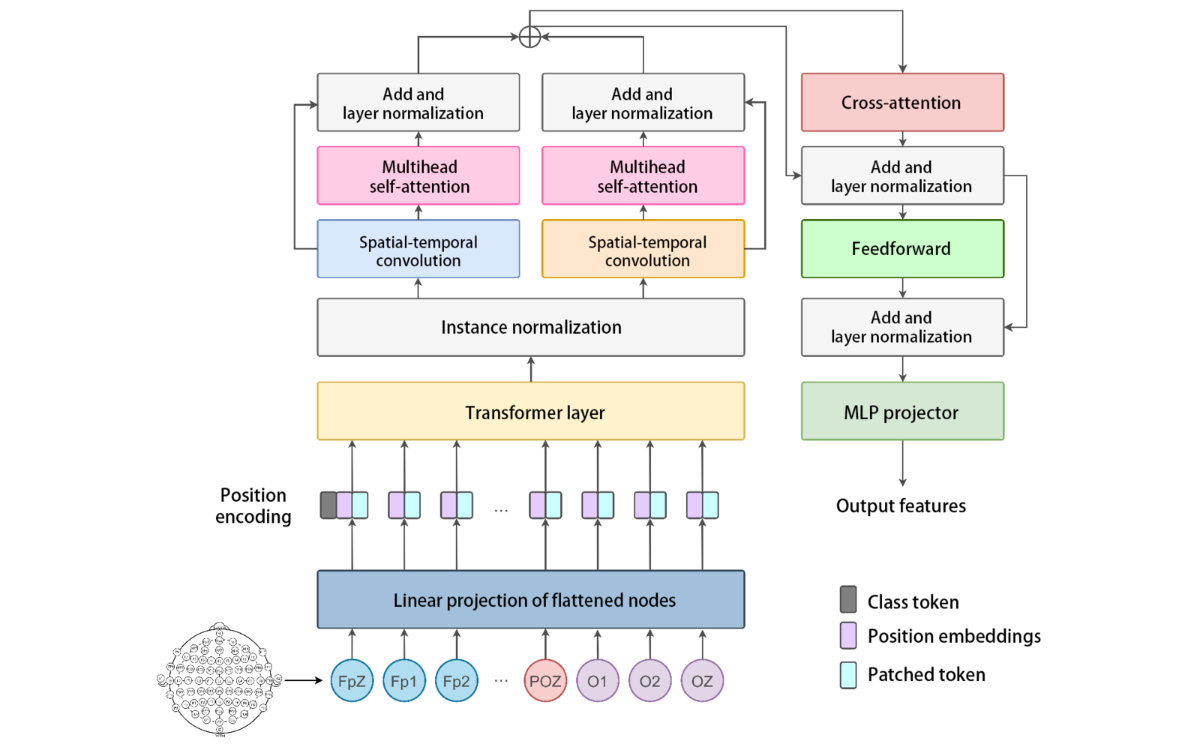
The overall structure of the Neural Encoding Representation Vectorizer electroencephalography encoder model. Fp1: frontal pole 1; Fp2: frontal pole 2; FpZ: frontal pole Z; MLP: multilayer perceptron; O1: occipital 1; O2: occipital 2; OZ: occipital midline; POZ: parieto-occipital midline.

As the foundational component of the NECOMIMI framework, we first describe our novel EEG encoder, NERV, which served as the foundation for EEG signal representation and embedding. As illustrated in Figure 2, NERV was designed to address the unique spatiotemporal complexity of EEG signals, which differ from natural images or text due to their nonstationary nature, simultaneous temporal dynamics, and spatial dependencies across multiple electrodes. To effectively capture these characteristics, NERV adopted a hierarchical architecture that combined convolutional and attention-based modules. The input EEG data, flattened and linearly projected, first underwent positional encoding to preserve temporal order. It then flowed through 2 complementary convolutional branches: a *spatial-temporal convolution*, which extracted spatial features followed by temporal ones, and a *temporal-spatial convolution*, which performed the reverse. These dual-pathway 2D convolutions operated across the EEG channel dimension, allowing the model to learn localized patterns in both directions. This design enabled NERV to generate robust and semantically meaningful embeddings from EEG signals, laying the groundwork for high-quality image generation in the NECOMIMI pipeline.

The output of these branches was passed through multihead self-attention layers, allowing the model to capture long-range dependencies across time and channel dimensions. Unlike conventional EEG encoders that treat all channels uniformly, NERV incorporated an EEG attention module at an early stage. This module used a transformer encoder across the channel axis to explicitly model interchannel relationships and assign adaptive importance weights to different brain regions. Such a design leveraged the structured spatial geometry of EEG electrode placement, enabling the model to generalize more effectively across participants. To further enhance discriminability, particularly in zero-shot settings, a cross-attention block was incorporated, enabling the class token to selectively attend to latent spatial-temporal features. The resulting feature sequence was flattened and passed through a fully connected layer, yielding a 1440D intermediate representation. Following this, a participant-specific linear transformation adjusted the temporal resolution to accommodate interindividual variability in EEG patterns. Finally, a residual projection head, composed of a Gaussian error linear unit activation and dropout, mapped the features into a 1024D latent space, providing a compact yet expressive representation for the downstream generative module.

The model was trained using a contrastive learning objective, aligning EEG embeddings with paired image embeddings from a pretrained CLIP model. This approach enabled robust multimodal representation learning, bridging neural signals and visual semantics. By combining structured convolution, channel-aware attention, and semantic alignment mechanisms, the NERV encoder effectively captured both the signal dynamics and spatial layout of EEG signals, significantly outperforming previous models in low-data and cross-participant scenarios. The EEG attention module explicitly modeled interchannel relationships and assigned adaptive importance to each channel based on its functional relevance, ensuring that different EEG channels are weighted according to their significance in the image generation process. By adopting this approach, NERV can effectively capture the unique spatial dependencies inherent in EEG data, leading to more accurate and contextually relevant representations for downstream image generation tasks.

### Framework Architecture and Experiment Setting Details

To ensure a fair comparison of various EEG encoders and minimize external factors, all experiments in this study were rerun using a unified CLIP–vision transformer (ViT) environment, leveraging the available open-source code [30-32]. CLIP is a deep learning model by OpenAI that uses contrastive learning to map both image and text into a shared embedding space, enabling the model to understand their relationship. It trains separate image and text encoders and supports zero-shot tasks such as image retrieval based on text. CLIP-ViT is a variant that uses ViT as the image encoder, which improves performance by processing images as patches. Given that contrastive learning is sensitive to batch size, a batch size of 1024 was used for all experiments. The final results were averaged across the best outcomes from 5 random-seed training sessions, each running for 200 epochs. We used the AdamW optimizer (The PyTorch Foundation) with a learning rate of 0.0002, and the β1 and β2 parameters were set to 0.5 and 0.999, respectively. The contrastive learning parameter τ was initialized with log(1/0.07). In terms of model architecture, the NERV model performed optimally with 5 multiheads, whereas the transformer layer used 1 multihead and the cross-attention layer used 8 multiheads. The time step for the diffusion model was set to 50. All experiments, including EEG encoder training and prior diffusion model processing, were conducted on a machine equipped with an A100 graphics processing unit.

Using the ThingsEEG dataset, we developed an advanced EEG-to-image generation model that leverages deep learning techniques and diffusion models. Initially, the framework included a 1-stage image generation phase. However, we observed that its performance was suboptimal. As a result, we adapted the model to a 2-stage process. The overall structure of the model was composed of 4 phases: training, zero-shot testing, 1-stage image generation, and 2-stage image generation (Figure 1). Each phase played a crucial role in transforming raw EEG data into meaningful visual outputs.

### Diffusion Model Implementation Details

To enhance the reproducibility and transparency of our EEG-to-image generation pipeline, we further describe the technical configuration of the diffusion model used in both the 1-stage and 2-stage NECOMIMI frameworks. We adopted the Stable Diffusion XL (SDXL) Turbo (Stability AI) [42,43] as the backbone generative model. SDXL Turbo is an optimized variant of the original Stable Diffusion architecture designed to produce high-quality images using significantly fewer sampling steps. This model is based on a U-Net structure with cross-attention mechanisms and operates in the latent space of a VAE, allowing for efficient and photorealistic image generation. For the denoising process, we applied a cosine noise schedule [14]. This schedule gradually adjusted the amount of noise during reverse diffusion, which helped improve image diversity and ensured smoother training convergence by avoiding sudden changes in noise levels across time steps. During inference, we used 50 sampling steps, which provided a good balance between generation speed and visual quality. This step count was well suited to SDXL Turbo’s low-latency design and has been empirically verified to maintain satisfactory image fidelity.

To condition the image generation on EEG signals, we used the IP-Adapter module [44]. Although originally developed for visual feature prompts, we adapted it to accept EEG embeddings. These embeddings were projected into the appropriate feature space and injected into the cross-attention layers of the U-Net at both low and middle resolutions, enabling the EEG signals to influence multiple stages of the generation process. Furthermore, we applied a classifier-free guidance strategy during image generation. This involved generating both conditional and unconditional outputs and blending them in a controlled manner to improve the relevance of the generated images to the input EEG signal while still preserving sample diversity. In our implementation, the guidance strength was set to a moderate value to maintain a balance between controllability and visual variation. In the 2-stage generation framework, the EEG embeddings were first processed by a lightweight diffusion prior network. This network, implemented as a 6-layer U-Net, was trained to transform noisy EEG embeddings into clean, CLIP-like image embeddings [30-32]. A dropout rate of 0.1 was used during training to encourage robustness. The resulting embeddings were then passed through the IP-Adapter into the SDXL Turbo model for final image synthesis. By integrating these components, the NECOMIMI framework effectively bridges the gap between EEG signals and generative models, enabling high-quality image synthesis guided purely by brain activity.

### Training Phase

In the initial training phase, both visual image ∈ 
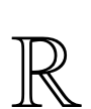
.*^h × w × ch^* and EEG data ∈ 
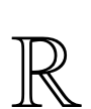
.*^e × d^* were processed in parallel to establish a shared embedding space. Here, ℎ denotes the image height, *w* is the image width, and *ch* refers to the number of channels (eg, RGB channels). Similarly, *e* represents the number of electrodes (channels), and *d* corresponds to the number of data points (time samples) in the EEG signal. Training set images were initially passed through a pretrained image encoder, which transforms the images into latent representations, referred to as image embeddings *I*. In this work, we used a pretrained ViT [45] from the CLIP model [28] as the image encoder, producing embeddings of size 
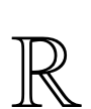
.^1 × 1024^ for each image. Simultaneously, the corresponding EEG signals are processed by a custom EEG encoder, generating EEG embeddings *E*. For the EEG encoder, this work extended several existing approaches, including NICE, MUSE, Nervformer, and Adaptive Thinking Mapper, to enable EEG-to-image capabilities [30-32]. Moreover, we introduced a novel EEG encoder, NERV, specifically designed for noisy, multichannel time-series data such as EEG that leveraged a multi-attention mechanism. The image and EEG embeddings were projected into a shared latent space using a multilayer perceptron projector, where they were optimized using the information noise-contrastive estimation (InfoNCE) loss function. This contrastive learning loss function ensured the alignment of corresponding image and EEG embeddings in the latent space, thereby improving the model’s capacity to associate neural patterns with visual stimuli. The InfoNCE loss, commonly used in contrastive learning, is defined as follows [28,46,47]: 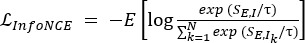. (1), where *S_E,I_* represents the similarity score between the EEG embeddings *E* and the paired image embeddings *I* and τ is the learned temperature parameter.

### Zero-Shot Testing Phase

Once trained, the model proceeded to the zero-shot testing phase, which evaluated its ability to generalize to unseen data. During this phase, the EEG signals and images from the test set were encoded using the pretrained encoders, and their respective embeddings were projected through the multilayer perceptron projector. The test data were divided into several groups, including 2-way, 4-way, 10-way, 50-way, and 100-way classification, facilitating a structured comparison between the EEG and image embeddings. The final similarity scores between the embeddings determine the model’s classification accuracy, enabling an evaluation of how well the model generalizes to new EEG data without requiring additional training.

### One-Stage Image Generation

In the 1-stage image generation process, EEG embeddings from the test set were directly used as inputs to reconstruct images. By incorporating the IP-Adapter [44], originally designed to use images as prompts, the compact design enhanced the flexibility of image prompts within pretrained text-to-image models. In this study, we adapted the IP-Adapter to transform EEG embeddings into *feature prompts* for the image generation process. These vectors were injected into the cross-attention layers of the U-Net at both low- and midresolution blocks, enabling the EEG embedding to modulate generation across multiple stages. These conditioned embeddings were then processed by the SDXL Turbo model [42,43]. This approach offered a streamlined method for EEG-based image generation, relying on a single transformation stage to produce meaningful visual outputs from neural signals. The commencement of the EEG-conditioned diffusion phase was crucial for generating images from EEG data. During this phase, a classifier-free guidance method was used, which paired CLIP embeddings and EEG embeddings (*I* and *E*). Specifically, we generated both conditional and unconditional outputs and combined them using the following formula: 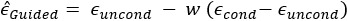. (2), where *w*≥0 is the guidance scale (set to 3.5 in our experiments). This technique improves controllability while preserving diversity.

By applying advanced generative techniques, the diffusion process was tailored to use the EEG embedding *E* to model the distribution of the CLIP embeddings *p*(*I*|*E*). The CLIP embedding *I*, generated in this stage, served as the foundation for the subsequent image generation phase. The architecture integrates a simplified U-Net model, denoted as ∈*_prior_* (*I^t^*, *t*, *E*), where *I^t^* represents the noisy CLIP embedding at a specific diffusion step *t*. The classifier-free guidance method refined the diffusion model by conditioning it on a specific EEG input *E*. This approach synchronized the outputs of both the conditional and unconditional models. The final model equation was expressed as follows: 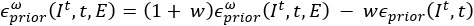. (3).

Where *w*≥0 was used to control the guidance scale, this technique allowed for training both the conditional and unconditional models within the same network, periodically replacing the EEG embedding *E* with a null value to introduce variation in the training process (approximately 10% of the data points). The primary goal was to improve the quality of the generated images while maintaining their diversity. However, we were surprised to find that when EEG embeddings were directly used as prompts for the diffusion model, the generated images predominantly consisted of landscapes regardless of the intended category. As a result, we explored a 2-stage approach for image generation.

### Two-Stage Image Generation

The prior diffusion stage played a crucial role in generating an intermediate representation [48], such as a CLIP image embedding, from a text caption [49]. This representation was then used by the diffusion decoder to produce the final image. This 2-stage process not only enhanced image diversity but also maintained photorealism, allowing for efficient and controlled image generation [50]. The 2-stage image generation process introduced a more sophisticated and refined method for synthesizing images from EEG data. In this approach, the EEG embeddings were initially processed by a diffusion prior network consisting of a 6-layer U-Net trained using cosine schedule and dropout (*P*=.10). After passing through the U-Net, the modified EEG embeddings were input into the SDXL Turbo model assisted by the IP-Adapter. This 2-step transformation ensured a more nuanced generation process, potentially leading to higher-quality images through deeper layers of refinement. The reverse diffusion process used a cosine noise schedule [42], which smoothly adjusts the variance over diffusion steps to stabilize training. This scheduling strategy improved sample diversity and convergence by avoiding abrupt noise scale changes across time steps. The time step *t*=50 referred to the number of reverse sampling steps used during inference. We found that 50 steps achieved a good trade-off between speed and quality, especially under the SDXL Turbo architecture, which was optimized for low-step generation.

The first step of stage 1 involved training the prior diffusion model. The primary goal of this training phase was to enable the model to recover the original embedding from noisy embeddings. The specific steps are detailed in Multimedia Appendix 1. Briefly, in the first phase of training, the model learned to reconstruct the original EEG embedding from a noisy version. Some EEG embeddings were randomly replaced with null values to introduce variability, and random noise was added and perturbed over multiple time steps. The model was trained to predict and remove this noise, effectively recovering the original EEG embedding. Once trained, the model entered the generation phase. It started with random noise, progressively refining it at each time step by predicting and removing noise. Both conditional and unconditional noise predictions were made and combined using a guidance mechanism to generate the final clean EEG embedding. In the second phase, the clean EEG embedding was passed through a specialized adapter, preparing it as a prompt for the image generation model. The final EEG embedding was then input into the SDXL Turbo model, which generated the final image based on the EEG data.

### Classification Task and Evaluation Strategy

We designed a participant-dependent zero-shot classification task to assess the semantic generalization ability of our EEG-to-image model. In this setting, the model was evaluated separately for each participant—a subset of image categories was used for training, whereas a disjoint set was held out for testing, ensuring a strict zero-shot scenario where the model must generalize to entirely unseen classes. Notably, no participant-specific adaptation methods (eg, participant embeddings or normalization layers) were used, and both training and testing were conducted on a within-subject basis. To align EEG and image features in a shared embedding space, we adopted the InfoNCE loss, a contrastive objective that encourages the model to pull matching EEG-image pairs closer while pushing apart nonmatching pairs within the same batch. This facilitates learning fine-grained semantic associations between brain activity and visual representations. We evaluated the model’s retrieval performance under zero-shot conditions using an *n*-way retrieval framework, where each test EEG sample was matched against *n* candidate image embeddings, including the correct one and *n* – 1 distractors sampled from unseen categories. A prediction was considered correct if the ground-truth image ranked highest in similarity. Retrieval difficulty was varied across 3 levels: 2-way task (1 distractor), 4-way task (3 distractors), and 200-way task (199 distractors from the full test set). This evaluation protocol provided a realistic and stringent benchmark for assessing semantic generalization and robust retrieval performance in EEG-to-image understanding.

### CAT Score

Unlike traditional image-to-image or text-to-image models driven by image representations, EEG-to-image models face unique challenges. In the current NECOMIMI architecture, the model can only capture broad semantic information from EEG signals rather than fine-grained details. For example, suppose the EEG data are recorded while a participant is viewing an aircraft carrier. When different EEG encoders are used in the NECOMIMI framework, they may lead to different outputs. Specifically, one EEG encoder could generate an image of a jet, whereas another EEG encoder might produce an image of a sheep. Therefore, to objectively assess performance, it was necessary to establish a standard that would score the encoder generating the jet image higher than the one generating the sheep image in such cases. Existing evaluation metrics were not suitable for this purpose. Traditional metrics such as the structural similarity index [51] measure structural similarity between the ground truth and the generated image, whereas the inception score [52] and Fréchet inception distance (FID) [53] focus on the accuracy of image categories and their distribution. However, EEG captures more abstract semantic information, and there was no guarantee that the participants’ thoughts during EEG recording would perfectly align with the ground-truth image. This made traditional evaluation methods unfair for EEG-to-image tasks. To address this issue, we proposed the CAT score, a new metric specifically designed for EEG-to-image evaluation (Multimedia Appendix 2). In the ThingsEEG test dataset, which contained 200 categories with 1 image per category, each image was manually labeled using 2 tags for broad categories, 1 for a specific category, 1 for background content, and 1 for object attribute, resulting in a total of 5 tags per image. The tags were extracted using ChatGPT-4o (OpenAI) [53]. The entire test dataset thus consisted of 200 images × 5 tags, totaling 1000 points. Using manual annotation, we were able to determine whether the categories of generated images matched these labels, providing a fair assessment for EEG-to-image models.

While FID remains a widely used metric for evaluating image fidelity by comparing feature distributions of generated and real images, it does not consider semantic alignment between the source EEG signal and the generated output [53]. In contrast, the CAT score directly evaluates whether the generated image aligns with the cognitive concept inferred from EEG signals. Therefore, the CAT score and FID served as complementary evaluation tools in our study. FID captures the visual quality, whereas the CAT score emphasizes semantic correctness, which is particularly crucial for EEG-driven generation where pixel-level supervision is unavailable. Further details on the ThingsEEG categories and scoring scheme are provided in Multimedia Appendix 2.

### Ethical Considerations

This study used publicly available datasets, and no personal or sensitive information was collected directly. All data were deidentified before analysis. As the study involved only publicly available data and did not involve the collection of personal data or clinical trials, institutional review board approval was not required per the Chang Gung University's Academic Ethics Series [54].

## Results

### Classification Results

Table 1 presents the classification accuracy results for the 2-way and 4-way zero-shot tasks across 10 participants. Our proposed model, NERV, consistently outperformed other methods, particularly excelling in the 2-way classification task, where it maintained the highest accuracy across most participants. Specifically, NERV achieved an average accuracy of 94.8% (SD 1.7%) in the 2-way task and 86.8% (SD 3.4%) in the 4-way task, surpassing competing models such as NICE, MUSE, and Adaptive Thinking Mapper ShallowNet (ATM-S). While NICE and MUSE demonstrated strong performance in certain participants, they fell short of NERV’s overall performance. NICE achieved an average accuracy of 91.3% (SD 3.1%) in the 2-way task and 81.3% (SD 5.9%) in the 4-way task, whereas MUSE achieved averages of 92.2% (SD 2.3%) and 82.8% (SD 5.0%), respectively. ATM-S, while comparable to NICE and MUSE in some participants, showed weaker results on average, with an accuracy of 86.5% (SD 4.9%) in the 4-way task. In the more challenging 200-way zero-shot classification task (Table 2), NERV also outperformed all other methods, particularly in terms of top-1 accuracy. Although ATM-S and NERV performed similarly overall, NERV demonstrated superior performance in most participants. NERV achieved an average top-1 accuracy of 27.9% (SD 5.8%) and an average top-5 accuracy of 54.7% (SD 6.5%), leading all other models. In contrast, Nervformer exhibited a significantly weaker performance, particularly in the top-1 accuracy, with averages of 19.8% (SD 4.9%) and 44.3% (SD 8.7%) for top-1 and top-5 accuracy, respectively. The results showed that NERV consistently outperformed its competitors across all tasks, demonstrating the strongest zero-shot classification performance, particularly in distinguishing between a large number of categories.

**Table 1:** Overall accuracy of 2-way and 4-way zero-shot classification using Contrastive Language-Image Pretraining–vision transformer as the image encoder. The average accuracy was reported along with the SD across 10 participants to reflect the statistical robustness of each method.

Method	Accuracy (%)																															Accuracy (%), mean (SD)	
	Participant 1			Participant 2			Participant 3			Participant 4			Participant 5			Participant 6			Participant 7			Participant 8			Participant 9			Participant 10					
	2-way task	4-way task	2-way task		4-way task	2-way task		4-way task	2-way task		4-way task	2-way task		4-way task	2-way task		4-way task	2-way task		4-way task	2-way task		4-way task	2-way task		4-way task	2-way task		4-way task	2-way task			4-way task
Nervformer	89.9	76.9	91.3		80.7	91.6		80.8	94.3		85.9	86.3		70.4	91.1		82.5	92.5		81.6	96.2		88.3	92		83.7	92.4		83.1	91.8 (2.6)			81.4 (4.9)
NICE^a^	91.7	80.4	89.9		77.4	93.5		83.7	94		84.9	85.9		70.3	89.1		81.7	91.2		87.1	95.8		89.2	87.9		76.5	93.8		87.7	91.3 (3.1)			81.3 (5.9)
MUSE^b^	90.1	78.4	90.3		76.8	93.4		85.6	93.6		87.5	88.3		74.2	93.1		85.3	93.1		85.3	95.4		87.7	90.5		81.8	94.4		88.1	92.2 (2.3)			82.8 (5.0)
ATM-S^c^	94.8	84.9	93.5		86.3	95.3		89	95.9		87.3	90.8		78.5	94.1		85.2	94.2		97.1	96.6		92.9	94.1		86.8	94.7		87	94.4 (1.6)			86.5 (4.9)
NERV^d^ (ours)	95.3	85.7	96		88.8	95.9		91.2	95.8		87.4	90.8		80.4	93.6		85.4	94.7		86.2	96.8		92.3	94.4		84.2	94.8		87.6	*94.8 (1.7)* ^e^			*86.8 (3.4)*

^a^NICE: Neural Image Conditioning for Electroencephalography.

^b^MUSE: Multimodal Unsupervised Sensing Embeddings.

^c^ATM-S: Adaptive Thinking Mapper ShallowNet.

^d^NERV: Neural Encoding Representation Vectorizer.

^e^The values in italics represent the best results (participant dependent—training and testing on 1 participant).

**Table 2:** Overall accuracy of 200-way zero-shot classification using Contrastive Language-Image Pretraining–vision transformer as image encoder—top 1 and top 5. The SD provided a measure of variability and generalization across participants.

Method	Accuracy (%)																					Accuracy (%), mean (SD)
	Participant 1		Participant 2		Participant 3		Participant 4		Participant 5		Participant 6		Participant 7		Participant 8		Participant 9		Participant 10			
	Top 1	Top 5	Top 1	Top 5	Top 1	Top 5	Top 1	Top 5	Top 1	Top 5	Top 1	Top 5	Top 1	Top 5	Top 1	Top 5	Top 1	Top 5	Top 1	Top 5	Top 1	Top 5
Nervformer	15	36.7	15.6	40	19.7	44.9	23.3	54.4	13	29.1	18.9	42.2	19.5	42	30.3	60	20.1	46.3	22.9	47.1	19.8 (4.9)	44.3 (8.7)
NICE^a^	19.3	44.8	15.2	38.2	23.9	51.4	24.1	51.6	11	30.7	18.5	43.8	21	47.9	32.5	63.5	18.2	42.4	27.4	57.1	21.1 (6.2)	47.1 (9.4)
MUSE^b^	19.8	41.1	15.3	34.2	24.7	52.6	24.7	52.6	12.1	33.7	22.1	51.9	21	48.6	33.2	59.9	19.1	43	25	55.2	21.7 (5.8)	47.3 (8.9)
ATM-S^c^	25.8	54.1	24.6	52.6	28.4	62.9	25.9	57.8	16.2	41.9	21.2	53	25.9	57.2	37.9	71.1	26	53.9	30	60.9	26.2 (5.6)	*56.5 (7.7)* ^d^
NERV^e^ (ours)	25.4	51.2	24.1	51.1	28.6	53.9	30	58.4	19.3	43.9	24.9	52.3	26.1	51.6	40.8	67.4	27	55.2	32.3	61.6	*27.9 (5.8)*	54.7 (6.5)

^a^NICE: Neural Image Conditioning for Electroencephalography.

^b^MUSE: Multimodal Unsupervised Sensing Embeddings.

^c^ATM-S: Adaptive Thinking Mapper ShallowNet.

^d^The values in italics represent the best results (participant dependent—training and testing on 1 participant).

^e^NERV: Neural Encoding Representation Vectorizer.

### Performance Comparison of Different Generative Models

Table 3 shows the results of our newly proposed CAT score method, which quantified and evaluated the quality of EEG-generated images based on their alignment with semantic concepts rather than pixel-level details. The specific CAT score labels are provided in Multimedia Appendix 2. Interestingly, although our NERV method achieved SOTA performance on the CAT score, no EEG encoder surpassed a score of 500 out of a possible 1000 points in this evaluation. This result underscored both the rigor of the CAT score and the inherent challenges associated with the pure EEG-to-image generation task.

**Table 3:** Overall Category-Based Assessment Table (CAT) score out of 1000 of the Neural-Cognitive Multimodal Electroencephalography (EEG)–Informed Image EEG-to-image generation using several EEG encoders. Average accuracy with SD across 10 participants was reported to indicate statistical reliability.

EEG encoder	CAT score (higher is better)											CAT score, mean (SD)
	Participant 1	Participant 2	Participant 3	Participant 4	Participant 5	Participant 6	Participant 7	Participant 8	Participant 9	Participant 10		
Nervformer	432	457	429	454	475	463	404	438	427	410	438.9 (23.0)	
NICE^a^	426	456	445	447	411	454	438	443	426	429	437.5 (14.3)	
MUSE^b^	438	456	434	416	426	463	443	437	410	468	439.1 (19.1)	
ATM-S^c^	413	419	411	464	427	469	442	472	431	445	439.3 (22.9)	
NERV^d^ (ours)	445	436	432	456	438	466	410	437	433	444	439.7 (14.9)	

^a^NICE: Neural Image Conditioning for EEG.

^b^MUSE: Multimodal Unsupervised Sensing Embeddings.

^c^ATM-S: Adaptive Thinking Mapper ShallowNet.

^d^NERV: Neural Encoding Representation Vectorizer.

The FID results for EEG-to-image generation using the NECOMIMI framework with several SOTA EEG encoders are shown in Table 4. This metric evaluated the similarity between generated images and real ones based on deep feature distributions, with lower scores indicating better visual fidelity. To assess the robustness and consistency of each method, we reported the average FID alongside its SD across 10 participants. As expected, the “Pure Test Image” baseline, which did not involve EEG input, achieved an ideal FID of 106.1. Among EEG-based approaches, our proposed NERV (2-stage) model outperformed all competitors, with the lowest mean FID being 183.8 (SD 13.3), significantly surpassing other 2-stage pipelines such as NICE (mean 218.7,  SD 7.5) and ATM-S (mean 197.1,  SD 18.9). This consistent superiority suggested that NERV may be more effective in learning semantically aligned and visually coherent representations from EEG signals. Notably, even the best EEG-based models exhibited a noticeable gap from the ideal FID score, highlighting the intrinsic difficulty of reconstructing high-fidelity images solely from brain signals. These findings collectively validated the effectiveness of our NERV architecture in capturing and translating complex neural patterns into meaningful visual outputs while also revealing the need for continued improvements in EEG-based generative modeling.

**Table 4:** Overall Fréchet inception distance (FID) of the Neural-Cognitive Multimodal Electroencephalography (EEG)–Informed Image EEG-to-image generation using several EEG encoders. The average accuracy alongside its SD calculated across 10 participants was reported to demonstrate the consistency of each method.

EEG encoder and embedding type			FID (lower is better)																					FID, mean (SD)
			Participant 1		Participant 2		Participant 3		Participant 4		Participant 5		Participant 6		Participant 7		Participant 8		Participant 9		Participant 10			
Pure test image			106.1		106.1		106.1		106.1		106.1		106.1		106.1		106.1		106.1		106.1		106.1 (0.0)	
**Nervformer**																								
	1 stage	240.6		241.7		259.9		247.4		251.5		241.2		241.3		247.1		250.6		244.8		246.6 (6.1)		
	2 stages	198.3		201.4		193.9		192.8		206.4		202.2		198.5		202.2		191.9		210.8		199.8 (6.0)		
**NICE^a^**																								
	1 stage	238.1		235.3		242.3		241.5		248.4		243.5		224.6		262.4		243.6		237.2		241.7 (9.7)		
	2 stages	204.3		193.2		264.9		198.9		199.1		272.0		235.3		209.7		213.1		196.1		218.7 (28.9)		
**MUSE^b^**																								
	1 stage	231.9		246.6		247.0		257.7		236.2		243.1		243.3		250.8		243.0		245.0		244.5 (7.1)		
	2 stages	188.7		218.5		184.5		198.1		215.9		186.5		206.7		190.5		195.4		204.7		199.0 (12.1)		
**ATM-S^c^**																								
	1 stage	244.5		239.7		238.6		242.1		248.9		250.1		232.2		250.3		239.0		235.0		242.0 (6.3)		
	2 stages	174.9		194.9		191.3		183.0		194.9		202.7		201.5		179.4		242.4		206.1		197.1 (18.9)		
**NERV^d^ (ours)**																								
	1 stage	217.9		211.4		225.6		221.1		229.1		218.8		215.5		227.6		204.6		217.2		*218.9 (* *7.5* *)* ^*e*^		
	2 stages	169.5		189.4		167.2		175.8		191.6		198.3		187.2		176.2		208.1		174.4		*183.8 (* *13.3* *)*		

^a^NICE: Neural Image Conditioning for EEG.

^b^MUSE: Multimodal Unsupervised Sensing Embeddings.

^c^ATM-S: Adaptive Thinking Mapper ShallowNet.

^d^NERV: Neural Encoding Representation Vectorizer.

^e^The values in italics represent the best results (participant dependent—training and testing on 1 participant).

### Perturbation Study on Visual Cortex EEG Channels

We further conducted a perturbation study to investigate whether the model disproportionately relied on EEG signals from the visual cortex, particularly in generating scene-based images. In EEG analysis, the visual cortex corresponds to posterior regions of the scalp, particularly the occipital and parieto-occipital areas. Accordingly, we identified 6 key EEG channels: occipital 1, occipital 2, occipital midline, parieto-occipital 3, parieto-occipital 4, and parieto-occipital midline, which are commonly associated with visual perception and spatial integration. These channels are known to play critical roles in visual information processing. Occipital 1 and occipital 2 are located over the left and right occipital lobes; these channels are strongly associated with early-stage visual perception and processing. Occipital midline is situated along the midline of the occipital region; this channel captures central visual cortex activity and contributes to basic visual field representation. Parieto-occipital 3 and parieto-occipital 4 are positioned at the parieto-occipital junction; these channels reflect integrative processing of spatial and visual information such as visuospatial attention. Parieto-occipital midline is a midline parieto-occipital channel involved in high-level integration of visual input and attentional modulation. In this experiment, we replaced the signals from these 6 channels with Gaussian noise while keeping the remaining EEG data unchanged. This allowed us to isolate the contribution of visual cortex–related signals to the image generation process. We then evaluated the quality of the generated images using the FID. Table 5 shows a significant increase in FID scores compared to the unperturbed condition, confirming that the model heavily depends on signals from the visual cortex to generate coherent and semantically meaningful images. These findings highlight the spatial sensitivity of our EEG-to-image model and further validate its biological plausibility in capturing relevant neural representations for visual imagination.

**Table 5:** Fréchet inception distance (FID) of the Neural-Cognitive Multimodal Electroencephalography (EEG)–Informed Image EEG-to-image generation using Neural Encoding Representation Vectorizer (NERV) EEG encoders with and without perturbation applied to visual cortex EEG channels. The average accuracy alongside its SD calculated over 10 participants was reported to demonstrate the consistency of each method.

EEG encoder and embedding type			FID (lower is better)																				FID, mean SD)
			Participant 1		Participant 2		Participant 3		Participant 4		Participant 5		Participant 6		Participant 7		Participant 8		Participant 9		Participant 10		
Pure test image			106.1		106.1		106.1		106.1		106.1		106.1		106.1		106.1		106.1		106.1		106.1 (0.0)
**NERV with perturbation**																							
	1 stage	257.4		244.9		263.3		266.3		268.4		235.5		263.5		243.6		212.7		256.6		251.2 (17.4)	
	2 stages	205.3		240.5		262.5		267.5		224.4		223.3		247.7		225.7		263.5		245.2		240.6 (20.6)	
**NERV without perturbation**																							
	1 stage	217.9		211.4		225.6		221.1		229.1		218.8		215.5		227.6		204.6		217.2		*218.9 (* *7.5* *)* ^a^	
	2 stages	169.5		189.4		167.2		175.8		191.6		198.3		187.2		176.2		208.1		174.4		*183.8 (* *13.3* *)*	

^a^The values in italics represent the best results (participant dependent—training and testing on 1 participant).

### Findings on EEG-to-Image Generation

**Figure 3:**
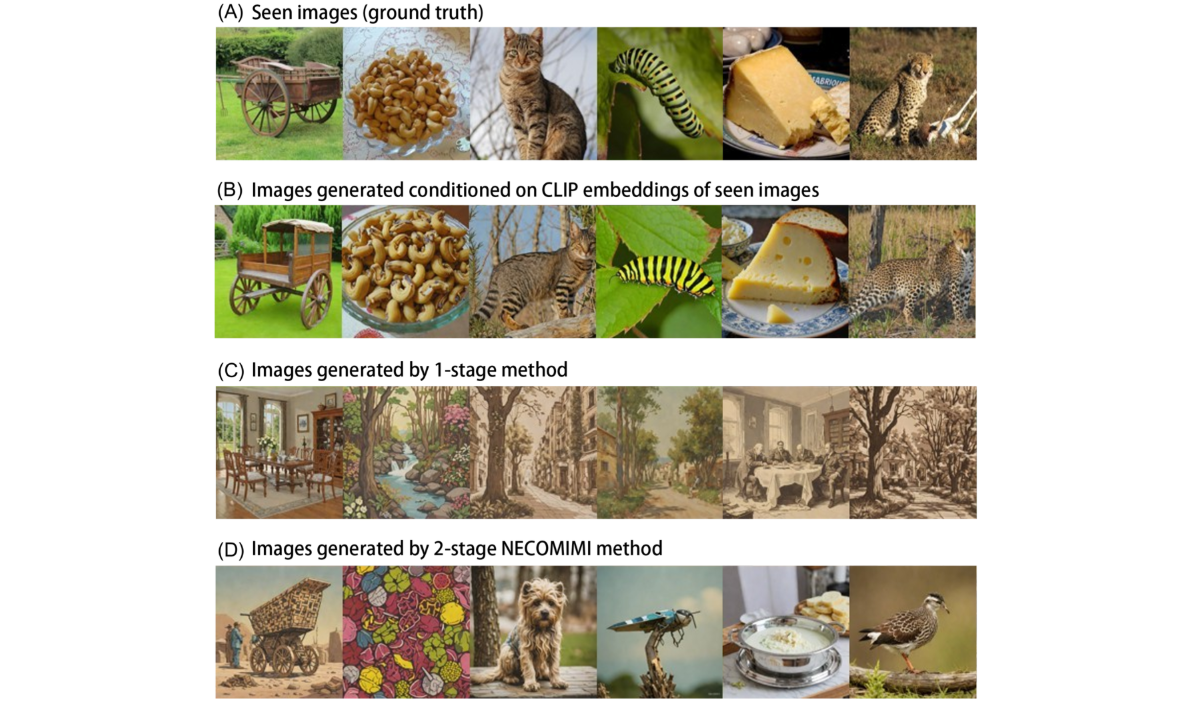
The progression of visual representations generated using different embedding techniques in a diffusion model—(A) the original images shown to participants during electroencephalography (EEG) recording (ground truth); (B) images generated using Contrastive Language-Image Pretraining (CLIP) embeddings of the ground-truth images serving as an upper-bound reference; (C) 1-stage Neural-Cognitive Multimodal EEG-Informed Image (NECOMIMI) results using EEG embeddings directly as prompts via the IP-Adapter; and (D) 2-stage NECOMIMI results, where EEG embeddings are refined into CLIP-like embeddings through a diffusion prior, producing images more consistent with object categories in the ground truth (eg, a cat in column 3 or a caterpillar in column 4).

**Figure 4:**
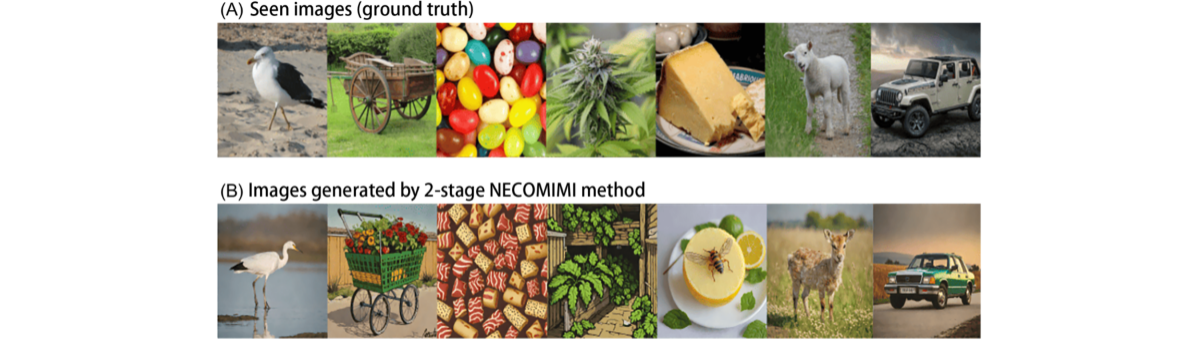
The capability of the Neural-Cognitive Multimodal Electroencephalography (EEG)–Informed Image (NECOMIMI) model to reconstruct images purely from EEG data without using the “Seen” images (ground truth) as embeddings during the generation process—(A) the original images shown to participants during EEG recording (ground truth) and (B) images generated using the 2-stage NECOMIMI method.

**Figure 5:**
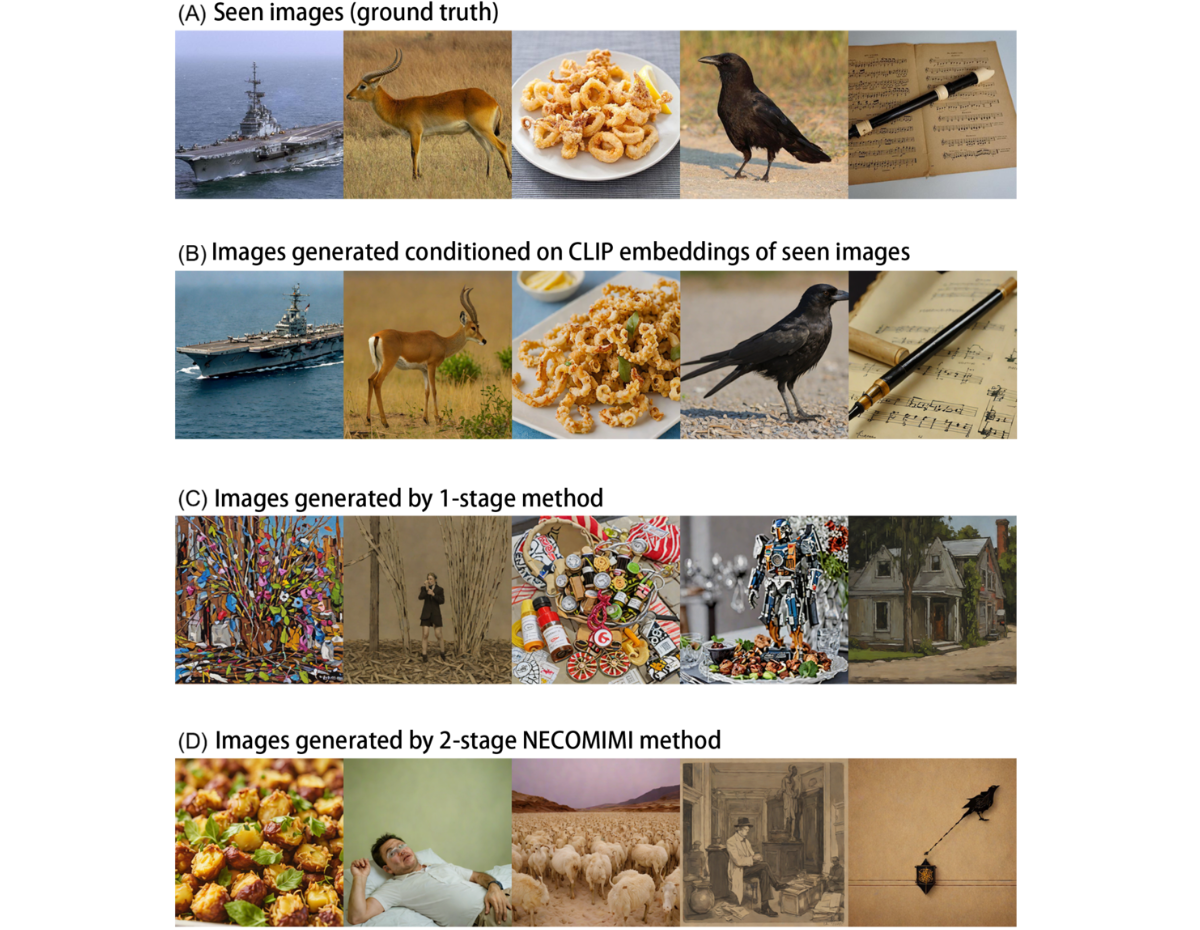
Neural-Cognitive Multimodal Electroencephalography (EEG)-Informed Image (NECOMIMI) EEG-to-image generation using Neural Encoding Representation Vectorizer EEG encoders with perturbation applied to visual cortex EEG channels—(A) the original images shown to participants during EEG recording (ground truth), (B) images generated conditioned on Contrastive Language-Image Pretraining (CLIP) embeddings of seen images, (C) images generated using the 1-stage NECOMIMI method, and (D) images generated using the 2-stage NECOMIMI method.

We observed several noteworthy findings in the pure EEG-to-image generation process (Figure 3). Figure 3A shows the original images shown to participants during EEG recording (ground truth). Figure 3B serves as an ideal baseline, demonstrating the model’s capacity when guided by semantically rich CLIP embeddings. As shown in Figure 3C, the images generated by the diffusion model from embeddings derived from EEG signals predominantly consisted of landscapes, which deviated substantially from the original (ground truth). Due to the inherently noisy and low-dimensional nature of EEG signals, these results often default to generic or landscapelike images, where outputs deviate significantly from the intended object categories. This suggested that EEG signals may not directly correspond to specific objects in images. As a result, the model tended to generate “safe options,” such as landscapes, which may have constituted a significant portion of the training data. This phenomenon, often referred to as “hallucination,” occurs when the model generates content that does not accurately represent the intended visual stimuli. Consequently, in the 1-stage image generation framework, EEG embeddings often led to abstract or generalized images such as landscapes rather than specific objects. In contrast, the 2-stage NECOMIMI architecture (Figure 3D) was able to effectively extract semantic information from noisy EEG signals. This additional transformation improved semantic alignment and supported more accurate object-level synthesis (eg, a cat in column 3 or a caterpillar in column 4). Nonetheless, failure cases still occurred, underscoring the challenge of decoding high-level visual semantics from EEG data. Overall, this figure shows the enhanced specificity and visual relevance achieved by the 2-stage pipeline compared to the 1-stage approach. Moreover, Figure 4 shows the NECOMIMI model’s ability to reconstruct images solely from EEG data without relying on “seen” images (ground truth) as embeddings during the generation process. The bottom row of images in Figure 4, generated exclusively from EEG input, highlights NECOMIMI’s potential to approximate the content of the “seen” images in the top row even without direct visual references or embeddings. Figure 5 illustrates the performance of NECOMIMI’s 1-stage and 2-stage EEG-to-image generation pipelines under perturbation of visual cortex channels. The reconstructed images show clear differences in visual fidelity and semantic alignment, emphasizing the model’s spatial sensitivity and its dependence on visual cortex activity for generating coherent visual representations.

## Discussion

### Principal Findings

In this study, our NERV EEG encoder demonstrated SOTA performance in various zero-shot classification tasks, including 2-way, 4-way, and 200-way classification, solidifying its role as an effective feature extraction tool for EEG signals. However, when applied in a 1-stage image generation framework where EEG embeddings from the encoder were directly used to generate images, the results often turned out to be abstract or overly generalized (eg, landscapes) rather than depicting specific objects. This suggests that EEG signals may reflect more abstract, high-level concepts or emotional responses related to viewing the images rather than concrete objects or specific details. These abstract concepts are likely associated with broader aspects of the scene or the brain’s generalized perception of the environment. Consequently, the model may be more inclined to generate generalized images such as landscapes than to focus on specific objects. This limitation underscores the inherent challenges of using EEG signals for precise image generation, primarily due to their noisy and low-resolution nature. Another contributing factor could be insufficient training of the model on EEG signals. The diffusion model might not yet be fully optimized to interpret and generate images from EEG data, particularly in cases in which the available data fail to adequately map EEG signals to specific visual representations. In addition, the significant gap between the visual modality and the neural modality (EEG) could further contribute to these challenges. Several factors may explain the noticeable discrepancies between the images generated from EEG signals and the ground truth, particularly in the failure to recognize specific objects.

Furthermore, this study highlights the critical role of visual cortex activity in EEG-based image generation. Our perturbation analysis, which involved replacing EEG signals from key occipital and parieto-occipital channels with Gaussian noise, led to a significant degradation in image quality, as reflected by increased FID scores. These results indicate that the NECOMIMI framework strongly depends on signals from visual cortical regions to generate semantically coherent images. This observation aligned with previous findings emphasizing the role of occipital areas in visual perception and EEG-based image reconstruction [55]. Importantly, this dependency also reveals potential limitations of such models in cases in which visual cortex function is impaired. Overall, these results further validate the biological grounding of our approach, demonstrating its ability to capture neural patterns linked to visual imagination.

To address these challenges, we proposed the 2-stage NECOMIMI framework, which combines the NERV EEG encoder with a diffusion-based generative model. The 2-stage NECOMIMI architecture effectively extracts semantic information from the noisy EEG signals, showing its ability to capture and represent underlying concepts derived from brain wave activity. In this framework, we introduced the CAT score, a new evaluation metric specifically designed for EEG-to-image generation. Unlike traditional image evaluation metrics, such as those that assess image distribution, the CAT score focuses on measuring the alignment between the generated images and the underlying semantic intent captured by EEG signals. This evaluation method provides a more accurate reflection of how well the generated images correspond to the cognitive state represented by the EEG data.

Interestingly, our analysis revealed a complex relationship between the encoder’s performance in classification tasks and the quality of the generated images. While a high-performing EEG encoder provides robust feature representations, the fidelity and specificity of the generated images were also influenced by other factors such as the generative model’s capacity to interpret the EEG embeddings. These findings underscore the challenges of bridging EEG signal processing with image generation and suggest that successful EEG-to-image translation requires both strong feature extraction and sophisticated generative modeling. Our perturbation study further reinforced the biological plausibility of the NECOMIMI framework. The model’s responsiveness to disruptions in specific EEG regions indicated its ability to extract spatially meaningful neural patterns, especially those critical for scene-level image synthesis. These results underscore the importance of spatial channel specificity and suggest that leveraging region-targeted EEG signals could further enhance generative performance in future applications.

While the primary focus of NECOMIMI was image generation, its potential extends far beyond this task, particularly in clinical applications for individuals with motor impairments. This section explores how NECOMIMI could be integrated into existing brain-computer interface (BCI) systems and enhanced assistive technologies. One of the applications of NECOMIMI is its ability to function as a visual feedback module in noninvasive BCI systems. Current BCIs for individuals with motor impairments primarily rely on classification-based intent detection methods, such as motor imagery or steady-state visual evoked potentials [56,57]. In contrast, NECOMIMI introduces a novel interaction modality, visual synthesis directly from thought. This approach could enable individuals with severe motor or speech impairments, such as those with locked-in syndrome, to communicate by visualizing concepts through their brain activity. Compared to traditional methods, this offers a more intuitive and flexible interface, allowing users to draw or visualize their thoughts, thereby enhancing their ability to express needs and emotions.

NECOMIMI could also play a critical role in neurofeedback therapies. In this context, real-time EEG-to-image generation may be used to reflect users’ mental states, such as emotions or imagined visual scenes, back to them as visual feedback. This feedback loop could be particularly beneficial in therapies targeting attention regulation, anxiety management, or exposure-based interventions [58]. By providing visual stimuli derived from brain activity, NECOMIMI may help users better understand and regulate their mental states, potentially leading to an effective therapeutic outcome. Moreover, NECOMIMI holds strong potential in the field of virtual rehabilitation. The framework could be integrated into immersive virtual environments where patients engage in therapy tasks. Rather than relying on conventional controllers, users could interact with the virtual world by generating images that represent specific tasks or intentions. This offers a more natural and intuitive input method, especially for users with motor disabilities who may struggle with standard navigation tools. EEG-driven image generation could enhance patient engagement and contribute to more personalized and effective rehabilitation processes. As the technology continues to evolve, integration with existing BCI platforms and assistive systems may broaden NECOMIMI’s impact, ultimately improving quality of life for individuals with motor and speech impairments. Although still in its early stages, EEG-driven image generation presents a promising direction for enriching user interaction in virtual rehabilitation. By enabling thought-driven image-based communication, NECOMIMI could lower the barrier to access for patients with limited motor control, paving the way for more inclusive and engaging therapy solutions.

Overall, our research presents a novel framework for EEG signal–to-image generation, which expands the application of existing BCI technologies and demonstrates its potential to improve quality of life for individuals with motor impairments. Traditional EEG-based control systems, particularly in clinical settings, often fail to meet expected outcomes. Although our study did not directly evaluate the effectiveness of EEG-controlled devices, it highlighted the promising clinical applications of EEG-to-image conversion. This technology could assist patients in controlling external devices (eg, prosthetics, wheelchairs, and smart environments) through EEG-generated images, thereby offering more opportunities for performing daily tasks and enhancing the accuracy and reliability of EEG-based control systems. Moreover, this approach allows for the transformation of brain sensory signals into realistic visual images, which could be presented in virtual environments, enabling patients to engage in visual perception and interaction based on EEG signals. This not only has the potential to enhance motivation and engagement during the treatment process but also offers possible benefits for rehabilitation therapy and anxiety management. Despite the significant clinical potential, the technology faces several challenges. Future research will need to address how to extract accurate and specific control commands from low-resolution, noise-prone EEG signals while ensuring their stability and reliability in real-world applications.

### Understanding the CAT Score Ceiling

The moderate CAT score observed across all EEG encoders, with no model exceeding a score of 500 out of 1000, raised an important question about the underlying limitations of EEG-to-image generation. This result reflected both inherent challenges of the EEG modality and technological constraints in current generative modeling approaches. Fundamentally, EEG signals are characterized by low spatial resolution, high temporal variability, and a strong susceptibility to noise, making it difficult to extract detailed, object-specific information from brain activity. Compared to neuroimaging modalities such as fMRI, EEG primarily captures global cortical dynamics and abstract cognitive states, which limits its capacity to convey fine-grained visual representations. From a technical perspective, existing diffusion models, even those enhanced with learned priors, are not fully adapted to decode such noisy and semantically diffuse embeddings. The reliance on vision language models such as CLIP may introduce additional complications as these models were trained on natural images and text, not on neural signals, leading to a mismatch in modality and representation [28]. The observed plateau around a CAT score of 500 was largely due to the structure of the scoring system, where each image was evaluated based on 5 semantic tags. Because the EEG embeddings often only weakly reflected specific visual features, the generated images typically aligned with only 1 to 3 of the intended tags. This partial semantic alignment resulted in midrange scores, often between 400 and 500. Furthermore, the generation outputs frequently exhibited generic or overly broad content such as landscapes or blurred objects, reflecting the limited conditioning power of EEG inputs. To overcome these limitations and improve performance beyond this ceiling, several strategies can be considered. Incorporating multimodal physiological signals such as eye tracking or electromyography may provide complementary information that helps disambiguate user intent and improve semantic grounding. In addition, training generative models specifically on EEG-to-image tasks rather than relying on pretrained vision language priors could reduce the modality gap and enhance fidelity. Building large-scale EEG-specific datasets and pretraining encoders on diverse cognitive states may also improve generalization. These directions highlight the importance of tailoring both model architecture and data curation to the unique characteristics of neural signals and point toward a future where EEG-based generative systems can achieve more accurate and reliable visual reconstructions.

### Limitations

Despite the promising results of the NECOMIMI framework in EEG-to-image generation, several key limitations remain that must be addressed in future research. One of the primary challenges in EEG-to-image research is the scarcity of publicly available, large-scale EEG-image pair datasets. Unlike text-to-image tasks, which benefit from extensive datasets such as LAION-5B, EEG-to-image studies are constrained by the limited availability of paired EEG and image data. While the ThingsEEG dataset is one of the largest of its kind, it still lacks the diversity and scale necessary to train highly generalized EEG-to-image models. This limitation hinders benchmarking efforts and prevents the development of robust generative models capable of generalizing across different EEG recording conditions, participants, and experimental paradigms. In addition to the dataset scarcity, EEG recordings exhibit significant interparticipant variability, meaning that neural responses to identical visual stimuli can vary considerably between individuals. This variability complicates the generalization of models across different participants. Furthermore, EEG-based datasets are often collected under controlled experimental conditions, which limits their applicability to real-world scenarios. Consequently, without access to larger, more diverse datasets, EEG-to-image models remain constrained in their ability to generalize across a wide range of neural and visual domains.

Another challenge arises from the fact that, although our NERV EEG encoder demonstrated SOTA performance in zero-shot classification tasks, this high classification accuracy did not necessarily translate into higher-quality image generation. This suggests that feature representations optimized for classification tasks may not be equally effective for generative tasks. The gap between feature extraction and image generation remains an open challenge, indicating the need for future models to explore alternative training strategies that better align EEG embeddings with the generative process. Optimizing NECOMIMI for real-time image generation could unlock new possibilities for applications in BCIs and neurofeedback systems. Furthermore, expanding training datasets to include more diverse and larger-scale EEG recordings will be essential for improving model generalization. Incorporating additional data modalities such as eye tracking, electromyography, or functional near-infrared spectroscopy could also provide complementary information to enhance image generation. Finally, future work should aim to improve the model’s resilience to signal noise and variability, particularly in the visual cortex. Multimodal integration and training protocols that encourage distributed cortical representation may reduce the model’s dependency on any single brain region, ultimately enhancing stability, interpretability, and generalization across individuals and tasks.

### Conclusions

The NECOMIMI framework expands previous work on EEG-image pair contrastive learning classification by enabling image generation, filling a gap in previous research and opening up new possibilities for EEG applications. We introduced the SOTA EEG encoder NERV, which achieved top performance in 2-way, 4-way, and 200-way zero-shot classification tasks, as well as in the CAT score evaluation, demonstrating its effectiveness in EEG-based generative tasks. A key finding was that the model often generated abstract images rather than specific objects. This suggests that EEG data, being noisy and low resolution, captured broad semantic concepts rather than detailed visuals. The gap between neural signals and visual stimuli remained a challenge for precise image generation. In addition, we proposed the CAT score, a new metric tailored for EEG-to-image generation, and established its benchmark on the ThingsEEG dataset. Surprisingly, we found that EEG encoder performance may not strongly correlate with the quality of the generated images, providing new insights into the limitations and challenges of this task. NECOMIMI demonstrates the potential of EEG-to-image generation while highlighting the complexities of translating neural signals into accurate visual representations. Furthermore, the perturbation experiment revealed that the NECOMIMI model strongly depends on EEG signals from the visual cortex, particularly the occipital and parieto-occipital regions. This reliance not only supports the biological plausibility of our model’s design but also emphasizes the critical role of spatial EEG features in accurate image reconstruction. These insights may guide future improvements in region-aware modeling strategies for EEG-based generative tasks. Future work should prioritize improving EEG representation learning, refining generative modeling techniques, and developing more robust evaluation frameworks to enhance the reliability and realism of EEG-generated images.

## Appendix

Multimedia Appendix 1 The 2-stage image generation process.

Multimedia Appendix 2 Details of the category-based assessment table score.

## Abbreviations

ATM-S: Adaptive Thinking Mapper ShallowNet
BCI: brain-computer interface
CAT: Category-Based Assessment Table
CLIP: Contrastive Language-Image Pretraining
EEG: electroencephalography
FID: Fréchet inception distance
fMRI: functional magnetic resonance imaging
GAN: generative adversarial network
InfoNCE: information noise-contrastive estimation
MUSE: Multimodal Similarity-Keeping Contrastive Learning
NECOMIMI: Neural-Cognitive Multimodal Electroencephalography-Informed Image
NERV: Neural Encoding Representation Vectorizer
NICE: Natural Image Contrast Electroencephalography
SDXL: Stable Diffusion XL
SOTA: state of the art
VAE: variational autoencoder
ViT: vision transformer
