# Supplementary material for: Exploring the Potential of Electroencephalography Signal–Based Image Generation Using Diffusion Models: Integrative Framework Combining Mixed Methods and Multimodal Analysis
Source: JMIR Med Inform. 2025 Jun 25;13:e72027. doi: 10.2196/72027 (PMC12242056; doi:10.2196/72027)
Supplement: Multimedia Appendix 1 [file medinform_v13i1e72027_app1.pdf]

Appendix A

Two-Stage Image Generation

The two-stage image generation approach plays a critical role in improving the quality of EEG-based image synthesis. The first stage, known as the *prior diffusion stage*, is responsible for generating an intermediate representation, such as a CLIP image embedding, derived from EEG input. This representation serves as the foundation for the second stage, where the final image is synthesized using a diffusion-based generative model. By adopting this two-stage approach, the system enhances the diversity of generated images, ensures photorealism, and allows for more controlled and structured image synthesis.

In this process, EEG embeddings are first transformed using a *Diffusion U-Net*, which applies multiple transformations to refine the neural data representation. These modified EEG embeddings are then passed through *Stable Diffusion XL-Turbo*, assisted by the *IP-Adapter*, to improve conditioning and fine-tune the final output. This two-step transformation helps the model extract more meaningful features from EEG data, resulting in higher-quality images through deeper refinement layers.

Stage 0: Training the Prior Diffusion Model

The first stage focuses on training a prior diffusion model, which learns to reconstruct clean embeddings from noisy ones. The training process follows these key steps:

1. **Random Masking:** A portion (10%) of the EEG conditional embeddings c_{emb} are randomly replaced with `None` to introduce variability in training:
$$c_{\text{emb}} = \text{None}, \quad \text{if random()} < 0.1 \tag{1}$$
2. **Noise Addition:** Gaussian noise is added to the target embedding h_{emb}, and the diffusion scheduler perturbs it at a random timestep t:
$$\hat{h}_{\text{emb}}(t) = \mathcal{S}_{\text{add\_noise}}(h_{\text{emb}}, \epsilon, t) \tag{2}$$
where $\epsilon \sim \mathcal{N}(0, I)$ represents the added noise.
3. **Noise Prediction:** The model takes the noisy embedding $\hat{h}_{\text{emb}}(t)$ along with the conditional embedding c_{emb} and predicts the noise component:
$$\epsilon_{\text{pred}} = \mathcal{D}_{\text{prior}}(\hat{h}_{\text{emb}}(t), t, c_{\text{emb}}) \tag{3}$$
4. **Loss Calculation:** The predicted noise is compared with the actual noise using *Mean Squared Error (MSE)* as the loss function:
$$L = \frac{1}{N} \sum_{i=1}^{N} (\epsilon_{\text{pred}}(i) - \epsilon(i))^2 \tag{4}$$
5. **Backpropagation and Optimization:** The loss is used to update model parameters θ through gradient descent:
$$\theta \leftarrow \theta - \eta \nabla_{\theta} L \tag{5}$$
where η is the learning rate.

Stage 1: Generating the Prior Embedding

Once training is complete, the model enters the generation phase, where it denoises embeddings iteratively to produce a clean representation. The process involves:

1. **Sampling Timesteps:** A sequence of denoising timesteps T is selected:
$$\{t_1, t_2, ..., t_T\} \sim \mathcal{T}(T) \tag{6}$$
2. **Initializing Noise Embedding:** A random noise embedding h_T is sampled as the starting point:
$$h_T \sim \mathcal{N}(0, I) \tag{7}$$
3. **Iterative Denoising:** The model gradually removes noise using conditional and unconditional predictions, applying *classifier-free guidance* for better control:
$$\epsilon_{\text{pred}} = \epsilon_{\text{pred\_uncond}} + \alpha_{\text{guide}}(\epsilon_{\text{pred\_cond}} - \epsilon_{\text{pred\_uncond}}) \tag{8}$$
where α_{guide} adjusts the influence of the conditional embedding.

4. **Updating the Embedding:** The cleaned representation is refined at each timestep using a scheduler function:

$$h_{t-1} = \mathcal{S}_{\text{step}}(\epsilon_{\text{pred}}, t, h_t) \tag{9}$$

5. **Final EEG Embedding Output:** After full denoising, the final EEG-derived embedding is obtained:

$$h_{\text{output}} = h_{\text{generated}} \in \mathbb{R}^{1\times 1024} \tag{10}$$

Stage 2: Image Generation with Stable Diffusion

In the second stage, the final EEG embedding h_{output} is used as a *prompt* for the *IP-Adapter*, which conditions *Stable Diffusion XL-Turbo* to generate the final image. This step ensures that the model effectively translates the EEG signal into a visually meaningful output.
