# Supplementary material for: Exploring the Potential of Electroencephalography Signal–Based Image Generation Using Diffusion Models: Integrative Framework Combining Mixed Methods and Multimodal Analysis
Source: JMIR Med Inform. 2025 Jun 25;13:e72027. doi: 10.2196/72027 (PMC12242056; doi:10.2196/72027)
Supplement: Multimedia Appendix 2 [file medinform_v13i1e72027_app2.pdf]

Appendix B

Category-based Assessment Table (CAT) Score

To rigorously evaluate the semantic fidelity of EEG-to-image generation, we introduce the Category-based Assessment Table (CAT) Score, a metric specifically designed to overcome the limitations of traditional visual similarity metrics such as SSIM or FID, which are ill-suited for assessing the abstract, non-pixel-aligned nature of brain signal generation.

The CAT Score operates by comparing the generated images to their ground truth counterparts using semantic tags derived from human-aligned language understanding. For each image in the ThingsEEG test set (200 images total), we first employed ChatGPT-4o[1] to generate five hierarchical semantic tags per ground truth image. The prompt used was: "Please provide me with 5 one-word descriptions of the image, ranging from high level to low level." The tags included:

- Two superordinate categories (e.g., animal, vehicle),
- One basic-level object label (e.g., dog, car),
- One background descriptor (e.g., outdoor, grass),
- One object attribute or affordance (e.g., metallic, used for transportation).

Each EEG-generated image was then manually evaluated by two independent raters against these five semantic tags of its corresponding ground truth. A tag was marked as a match if the generated image clearly depicted or strongly implied the semantic concept. Disagreements were resolved via discussion, and importantly, no automated classifiers were used to avoid model-induced bias.

Scoring was conducted across all 200 image pairs, with 5 tags per image, resulting in 1,000 tag-match opportunities. One point was awarded for each correctly matched tag, yielding a maximum CAT Score of 1,000. This partial credit approach allows for nuanced quantification of semantic alignment, especially when the generation captures some, but not all, aspects of the target concept.

Image Label	Test Image in ThingsEEG	Category-based label
00001_aircraft_carrier	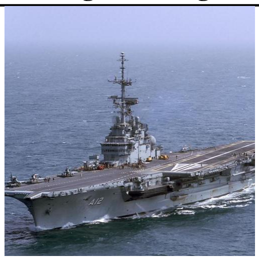	Ship Carrier Deck Island Antenna
00002_antelope	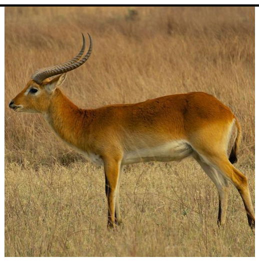	Animal Antelope Fur Grassland Horns

Continued on next page

[1] https://chatgpt.com

Image Label	Test Image in ThingsEEG	Category-based label
00003_backscratcher	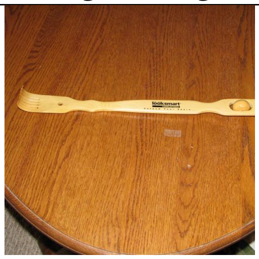	Object Tool Backscratcher Wood Handle
00004_balance_beam	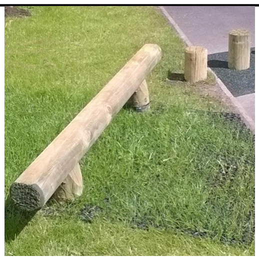	Structure Beam Wood Grass Support
00005_banana	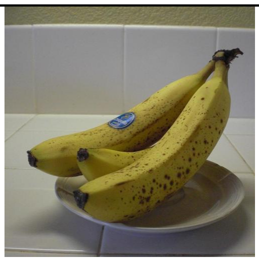	Fruit Banana Yellow Spotted Plate
00006_baseball_bat	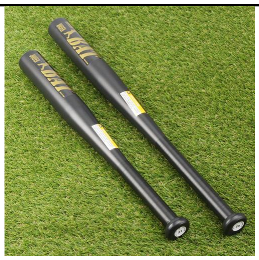	Sports Bats Baseball Black Grass
00007_basil	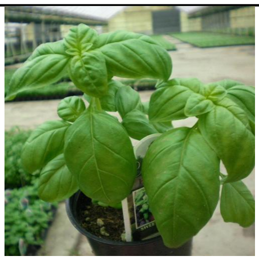	Plant Herb Basil Green Leaves
00008_basketball	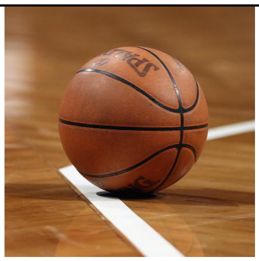	Sport Basketball Ball Orange Court

Continued on next page

Image Label	Test Image in ThingsEEG	Category-based label
00009_bassoon	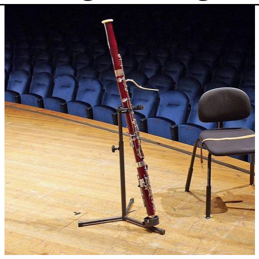	Instrument Bassoon Woodwind Stage Chair
00010_baton4	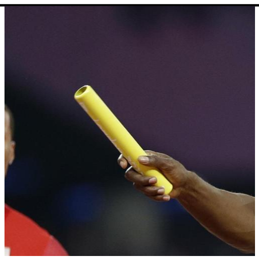	Race Relay Baton Yellow Hand
00011_batter	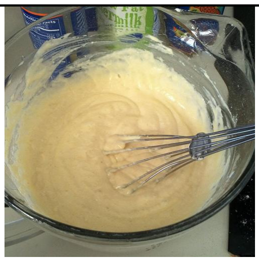	Cooking Batter Mixing Whisk Bowl
00012_beaver	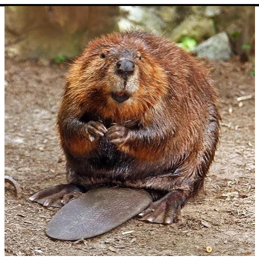	Animal Beaver Fur Tail Paws
00013_bench	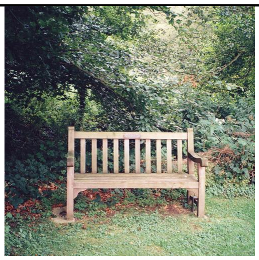	Outdoor Bench Wooden Garden Trees
00014_bike	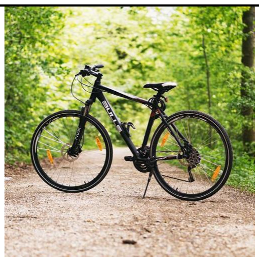	Bicycle Road Wheels Frame Path

Continued on next page

Image Label	Test Image in ThingsEEG	Category-based label
00015_birthday_cake	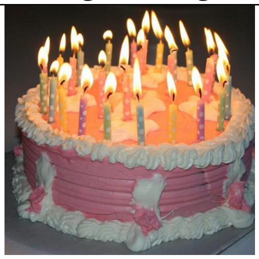	Cake Candles Flames Pink Frosting
00016_blowtorch	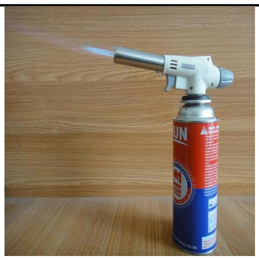	Tool Blowtorch Flame Canister Gas
00017_boat	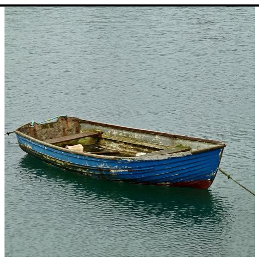	Boat Water Blue Old Rowing
00018_bok_choy	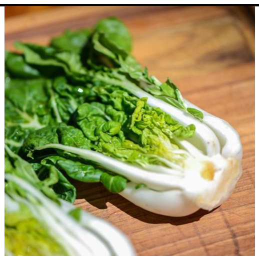	Vegetable BokChoy Green Leafy Stems
00019_bonnet	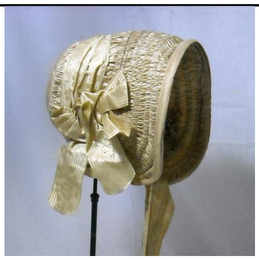	Hat Bonnet Ribbon Fabric Vintage
00020_bottle_opener	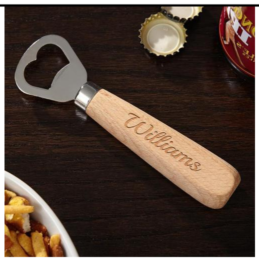	Tool Opener Wooden Bottlecap Engraving

Continued on next page

Image Label	Test Image in ThingsEEG	Category-based label
00021_brace	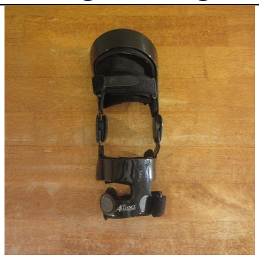	Support Brace Joint Black Strap
00022_bread	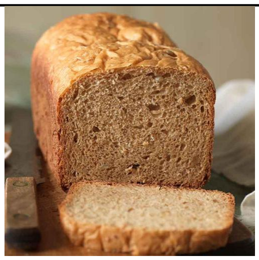	Food Bread Loaf Slice Crust
00023_breadbox	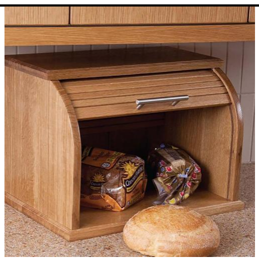	Storage Breadbox Wooden Bread Countertop
00024_bug	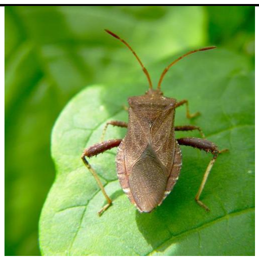	Insect Bug Leaf Brown Antennae
00025_buggy	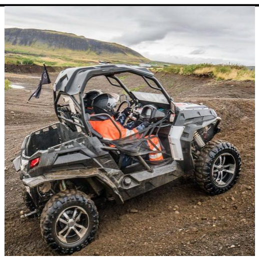	Vehicle Buggy Off-road Wheels Helmet
00026_bullet	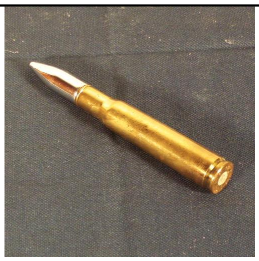	Ammunition Bullet Brass Cartridge Metal

Continued on next page

Image Label	Test Image in ThingsEEG	Category-based label
00027_bun	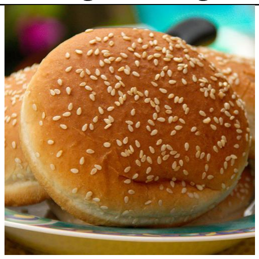	Food Bun Sesame Bread Round
00028_bush	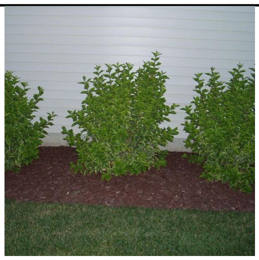	Plants Bushes Green Mulch Shrub
00029_calamari	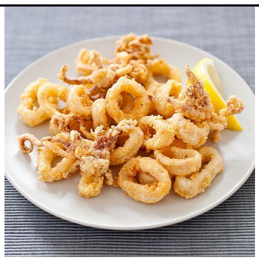	Food Calamari Fried Plate Lemon
00030_candlestick	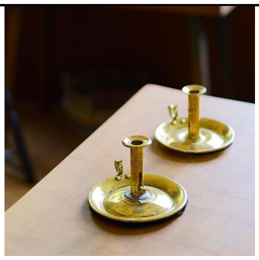	Candlesticks Brass Holders Antique Table
00031_cart	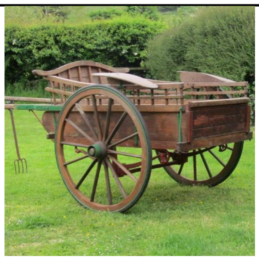	Cart Wheels Wooden Farm Grass
00032_cashew	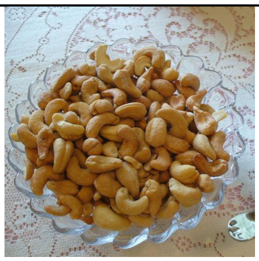	Nuts Cashews Bowl Snack Glass

Continued on next page

Image Label	Test Image in ThingsEEG	Category-based label
00033_cat	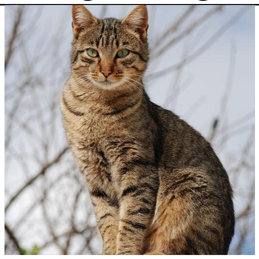	Animal Cat Tabby<br>Fur Whiskers
00034_caterpillar	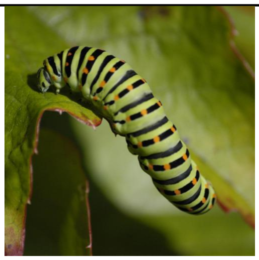	Insect Caterpillar Striped<br>Green Leaf
00035_cd_player	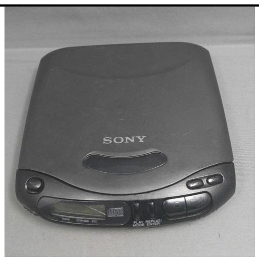	Device CDPlayer Portable<br>Gray Buttons
00036_chain	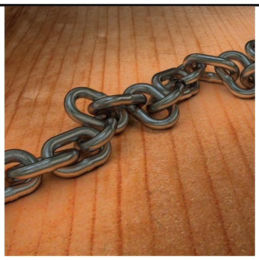	Metal Chain Links<br>Rusty Wood
00037_chaps	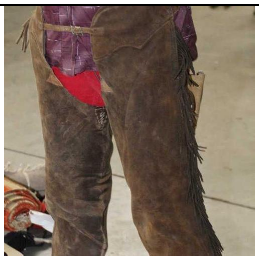	Clothing Chaps Leather<br>Fringe Brown
00038_cheese	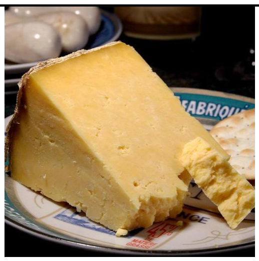	Food Cheese Wedge<br>Yellow Cracker

Continued on next page

Image Label	Test Image in ThingsEEG	Category-based label
00039_cheetah	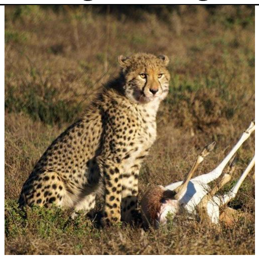	Animal Cheetah Spotted Hunt Grassland
00040_chest2	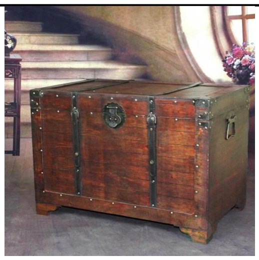	Furniture Chest Wooden Vintage Lock
00041_chime	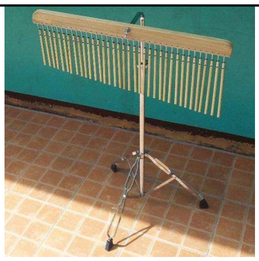	Instrument Chime Percussion Metal Stand
00042_chopsticks	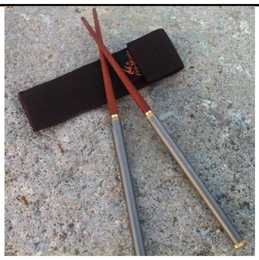	Utensils Chopsticks Wooden Metal Case
00043_cleat	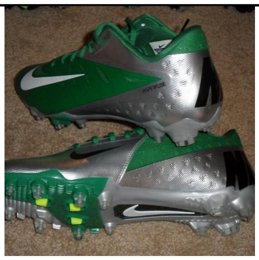	Footwear Cleats Shoe Green Studs
00044_cleaver	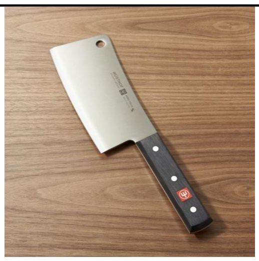	Tool Cleaver Blade Handle Steel

Continued on next page

Image Label	Test Image in ThingsEEG	Category-based label
00045_coat	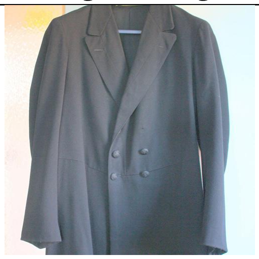	Clothing Coat Black Double-breasted Hanger
00046_cobra	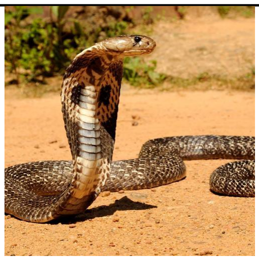	Animal Cobra Snake Hood Sand
00047_coconut	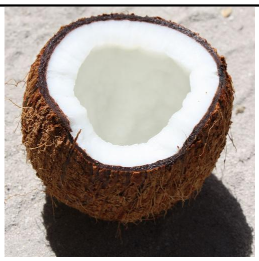	Fruit Coconut Shell White Husk
00048_coffee_bean	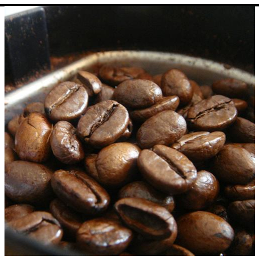	Coffee Beans Roasted Brown Grinder
00049_coffeemaker	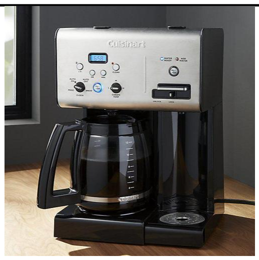	Appliance Coffeemaker Machine Carafe Buttons
00050_cookie	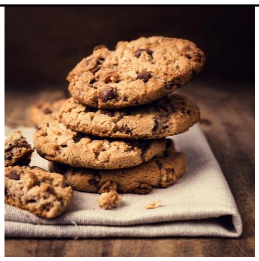	Cookies Snack Chocolate Stack Crumb

Continued on next page

Image Label	Test Image in ThingsEEG	Category-based label
00051_cordon_bleu	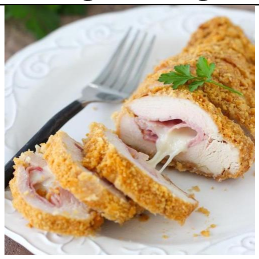	Food Chicken CordonBleu Breaded Stuffed
00052_coverall	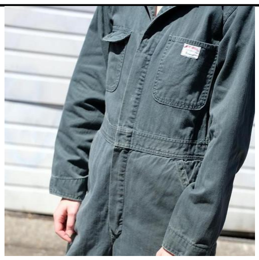	Clothing Coverall Workwear Pockets Green
00053_crab	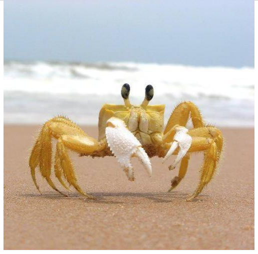	Animal Crab Beach Claws Sand
00054_creme_brulee	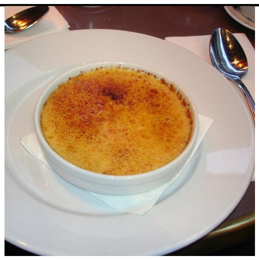	Dessert CrèmeBrûlée Caramelized Custard Spoon
00055_crepe	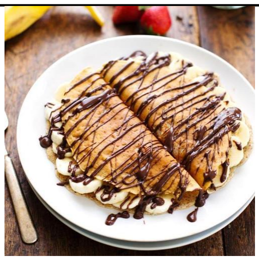	Dessert Crepe Chocolate Banana Plate
00056_crib	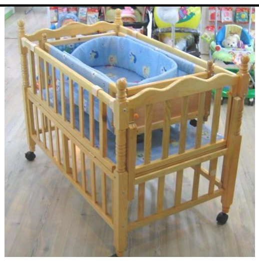	Furniture Crib Wooden Baby Bedding

Continued on next page

Image Label	Test Image in ThingsEEG	Category-based label
00057_croissant	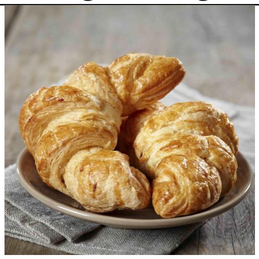	Pastry Croissant Flaky Golden Plate
00058_crow	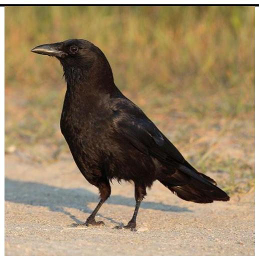	Bird Crow Black Feathers Beak
00059_cruise_ship	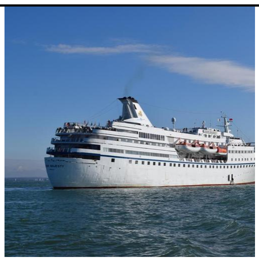	Vessel Cruise Ship Ocean Deck
00060_crumb	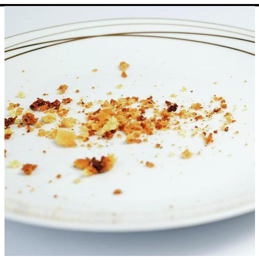	Crumbs Plate Food Leftovers White
00061_cupcake	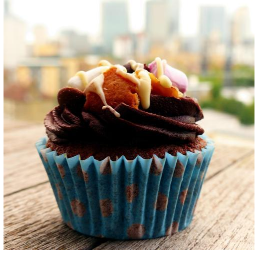	Cupcake Dessert Chocolate Icing Wrapper
00062_dagger	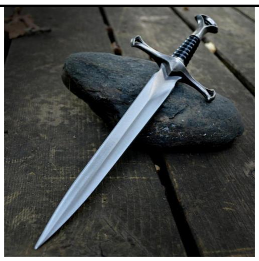	Weapon Dagger Blade Handle Rock

Continued on next page

Image Label	Test Image in ThingsEEG	Category-based label
00063_dalmatian	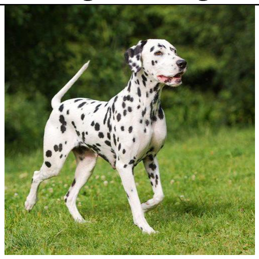	Dog Dalmatian Spotted White Grass
00064_dessert	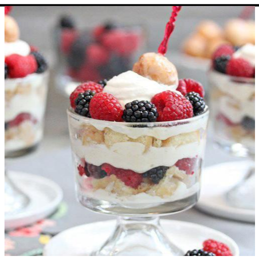	Dessert Berries Cream Trifle Glass
00065_dragonfly	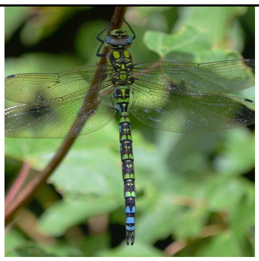	Insect Dragonfly Wings Striped Branch
00066_dreidel	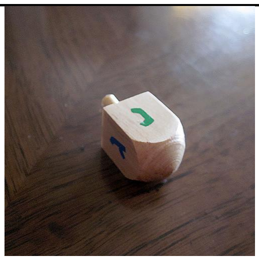	Toy Dreidel Wooden Spinning Letters
00067_drum	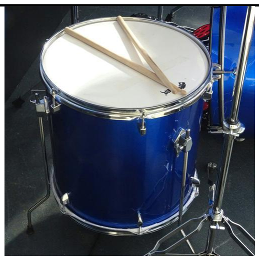	Instrument Drum Sticks Blue Percussion
00068_duffel_bag	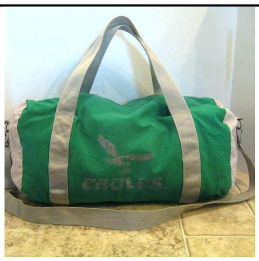	Bag Container Green Straps Eagles

Continued on next page

Image Label	Test Image in ThingsEEG	Category-based label
00069_eagle	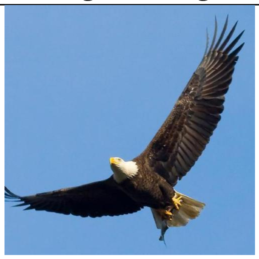	Bird Eagle Flight Wings Sky
00070_eel	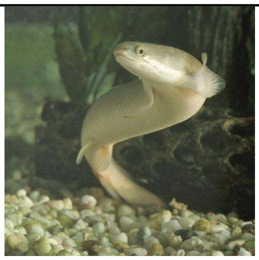	Fish Eel Aquatic Tank Gravel
00071_egg	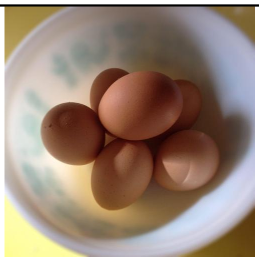	Eggs Bowl Brown Food Shell
00072_elephant	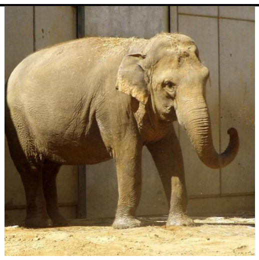	Animal Elephant Trunk Zoo Mammal
00073_espresso	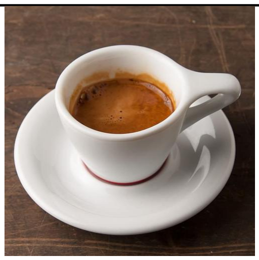	Drink Espresso Cup Coffee Saucer
00074_face_mask	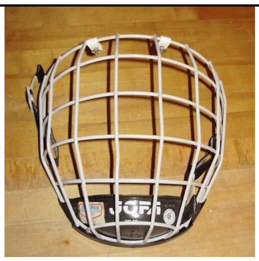	Gear Mask Helmet Cage Protection

Continued on next page

Image Label	Test Image in ThingsEEG	Category-based label
00075_ferry	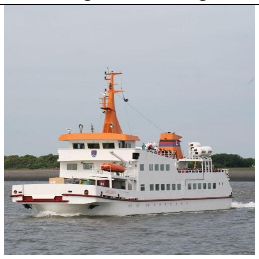	Ferry Boat Transport Water Orange
00076_flamingo	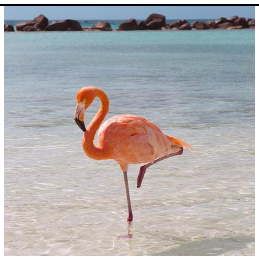	Bird Flamingo Pink Water Beach
00077_folder	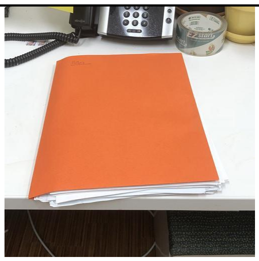	Folder Office Orange Papers Desk
00078_fork	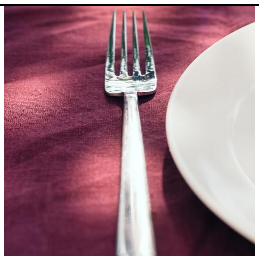	Utensil Fork Silver Plate Tablecloth
00079_freezer	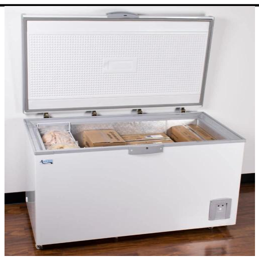	Appliance Freezer Storage Cold White
00080_french_horn	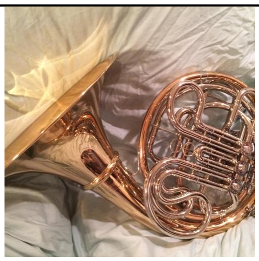	Instrument Horn Brass Coiled Shiny

Continued on next page

Image Label	Test Image in ThingsEEG	Category-based label
00081_fruit	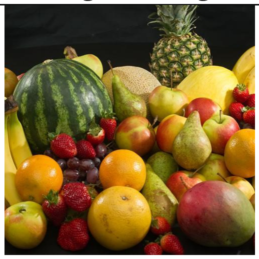	Fruits Assortment Tropical Colorful Fresh
00082_garlic	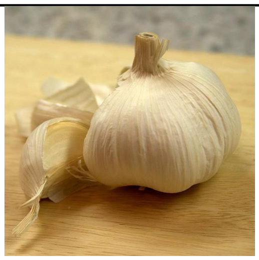	Garlic Bulb Cloves White Peeled
00083_glove	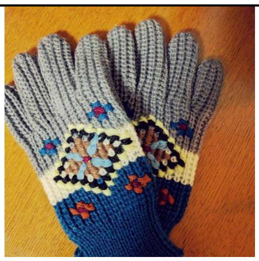	Gloves Knitted Patterned Wool Gray
00084_golf_cart	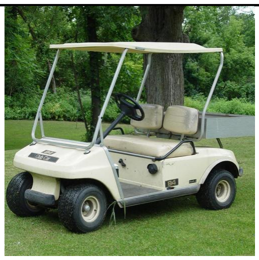	Vehicle GolfCart White Seats Wheels
00085_gondola	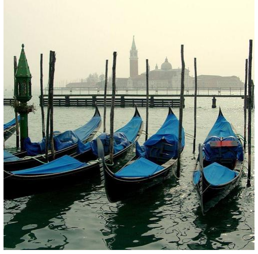	Boats Gondolas Venice Water Blue
00086_goose	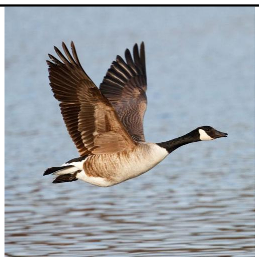	Bird Goose Flight Wings Lake

Continued on next page

Image Label	Test Image in ThingsEEG	Category-based label
00087_gopher	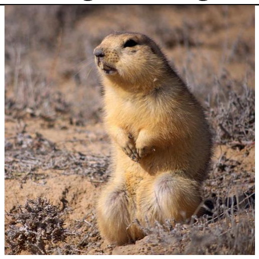	Animal Gopher Furry Rodent Field
00088_gorilla	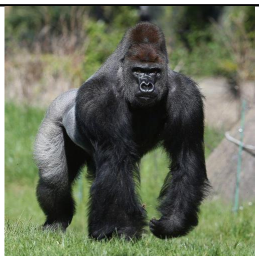	Animal Gorilla Primates Silverback Grass
00089_grasshopper	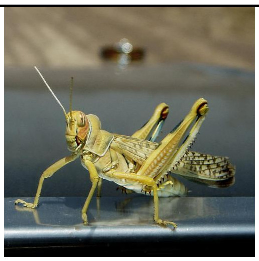	Insect Grasshopper Antennae Legs Green
00090_grenade	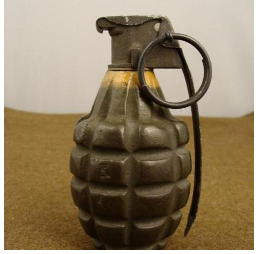	Weapon Grenade Metal Pin Explosive
00091_hamburger	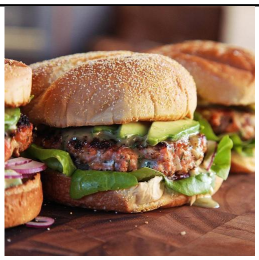	Food Hamburger Bun Lettuce Grilled
00092_hammer	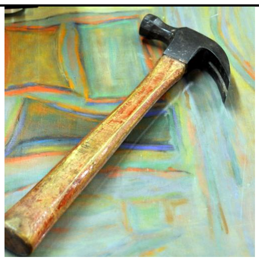	Tool Hammer Handle Metal Claw

Continued on next page

Image Label	Test Image in ThingsEEG	Category-based label
00093_handbrake	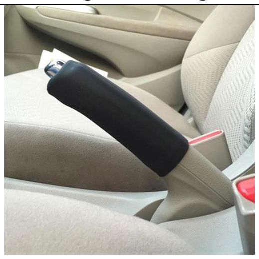	Automobile Interior Handbrake Lever Grip
00094_headscarf	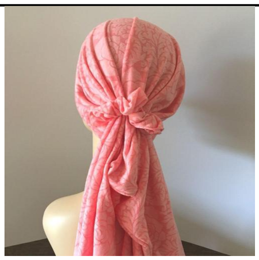	Headwear Scarf Fabric Pink Wrap
00095_highchair	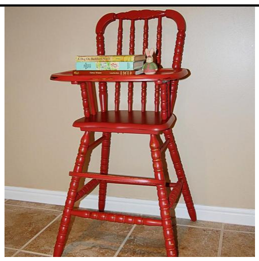	Red Wooden Chair Highchair Furniture
00096_hoodie	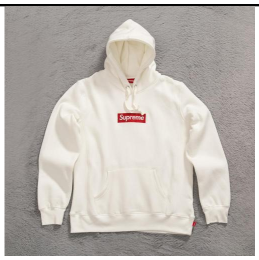	White Hoodie Ground Casual Clothing
00097_hummingbird	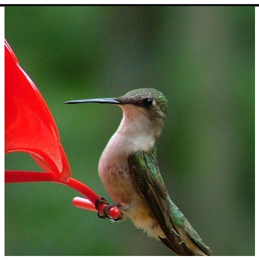	Hummingbird Green Feeder Small Bird
00098_ice_cube	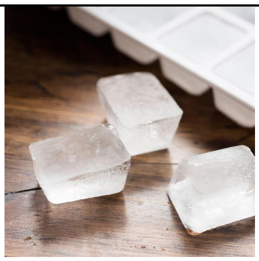	Ice Cold Frozen Clear Cubes

Continued on next page

Image Label	Test Image in ThingsEEG	Category-based label
00099_ice_pack	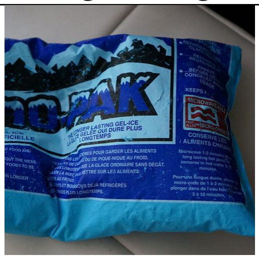	Gel Blue Reusable Cold Cooling
00100_jeep	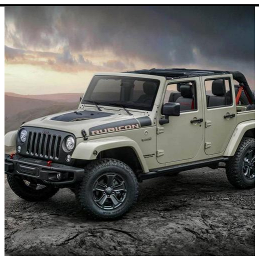	Off-road Rugged SUV Adventure Durable
00101_jelly_bean	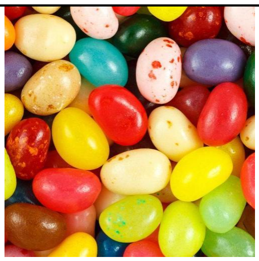	Colorful Sweet Candy Vibrant Chewy
00102_jukebox	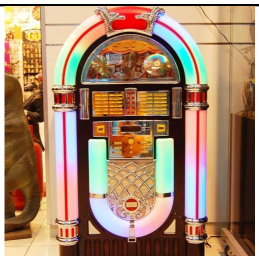	Retro Vibrant Music Neon Classic
00103_kettle	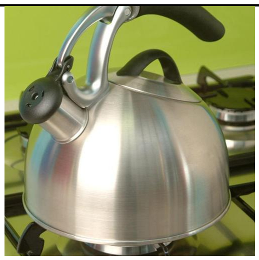	Shiny Stovetop Whistling Metallic Classic
00104_kneepad	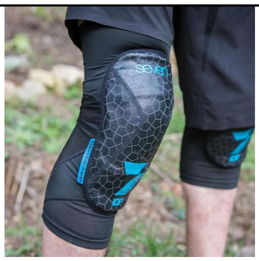	Protective Sporty Durable Cushioned Ergonomic

Continued on next page

Image Label	Test Image in ThingsEEG	Category-based label
00105_ladle	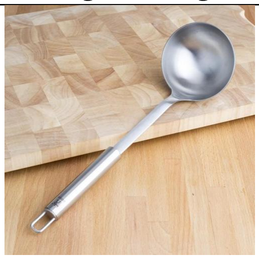	Stainless Sleek Functional Polished Culinary
00106_lamb	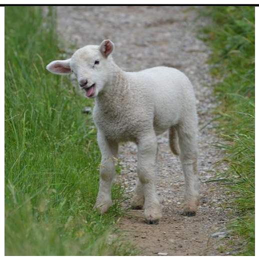	Adorable Fluffy Playful Animal Lamb
00107_lampshade	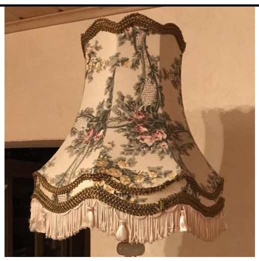	Vintage Floral Fabric Fringed Ornate
00108_laundry_basket	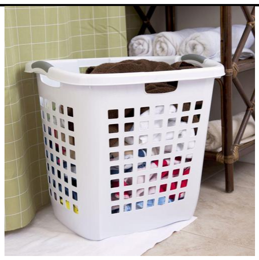	Laundry Plastic Basket Towels Grid
00109_lettuce	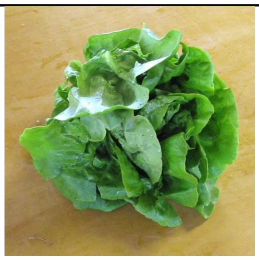	Vegetable Lettuce Leafy Fresh Green
00110_lightning_bug	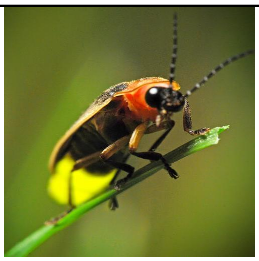	Insect Firefly Antennae Glowing Segmented

Continued on next page

Image Label	Test Image in ThingsEEG	Category-based label
00111_manatee	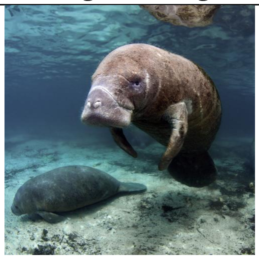	Aquatic Manatee Underwater Mammal Floating
00112_marijuana	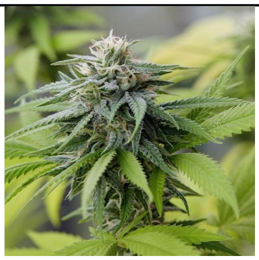	Cannabis Plant Buds Leaves Green
00113_meatloaf	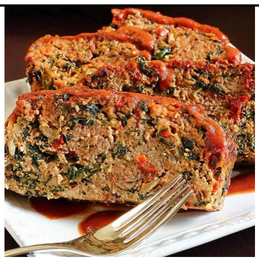	Food Meatloaf Slice Sauce Hearty
00114_metal_detector	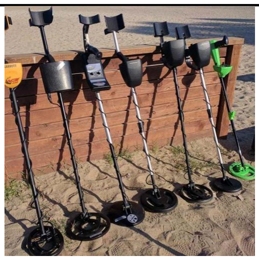	Equipment Detectors Metal Beach Lineup
00115_minivan	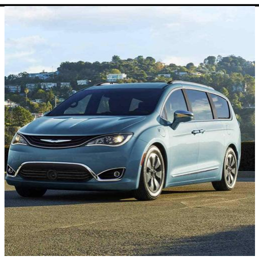	Vehicle Minivan Car Blue Electric
00116_modem	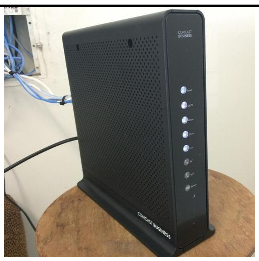	Device Modem Router Black Connectivity

Continued on next page

Image Label	Test Image in ThingsEEG	Category-based label
00117_mosquito	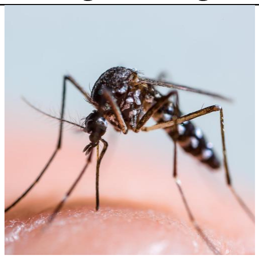	Insect Mosquito Biting Legs Proboscis
00118_muff	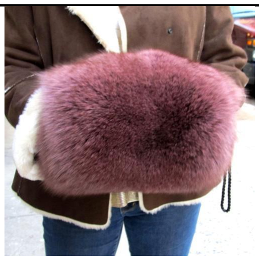	Accessory Muff Fur Warm Pink
00119_music_box	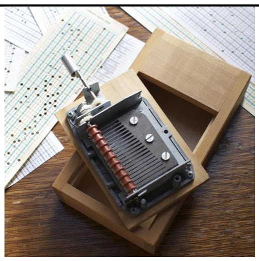	Device Music Box Crank Punched
00120_mussel	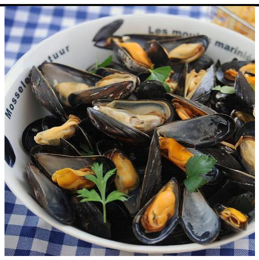	Seafood Mussels Shells Steamed Parsley
00121_nightstand	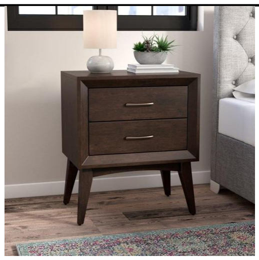	Furniture Nightstand Wooden Drawer Lamp
00122_okra	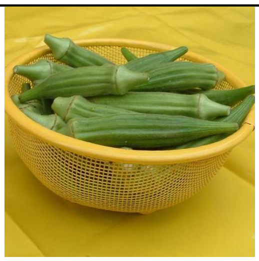	Vegetable Okra Green Basket Fresh

Continued on next page

Image Label	Test Image in ThingsEEG	Category-based label
00123_omelet	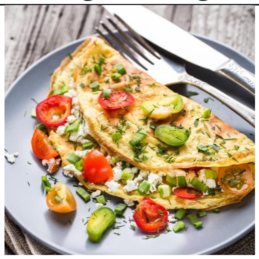	Breakfast Omelet Vegetables Tomatoes Herbs
00124_onion	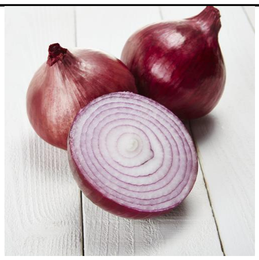	Vegetable Onion Red Sliced Raw
00125_orange	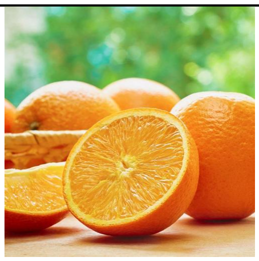	Fruit Orange Citrus Sliced Juicy
00126_orchid	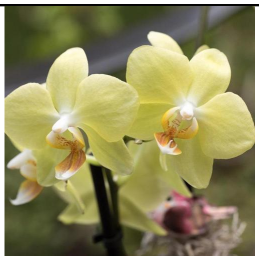	Flower Orchid Yellow Bloom Petals
00127_ostrich	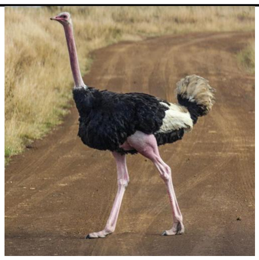	Bird Ostrich Large Plumage Road
00128_pajamas	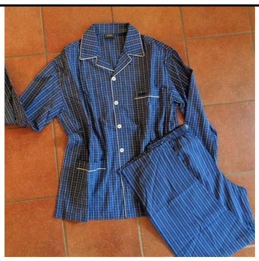	Clothing Pajamas Striped Blue Fabric

Continued on next page

Image Label	Test Image in ThingsEEG	Category-based label
00129_panther	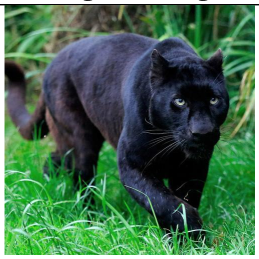	Animal Panther Black Predator Stealthy
00130_paperweight	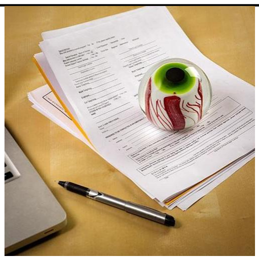	Office Paperwork Paperweight Eyeball Documents
00131_pear	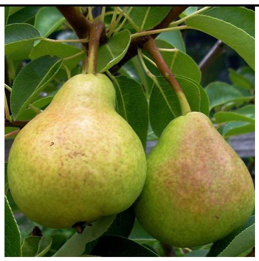	Fruit Pear Tree Green Ripe
00132_pepper1	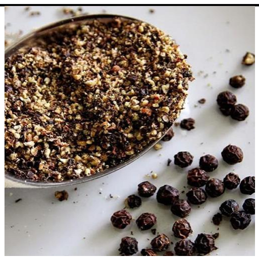	Spice Pepper Ground Black Spoon
00133_pheasant	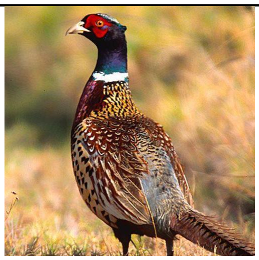	Bird Pheasant Feathers Colorful Wild
00134_pickax	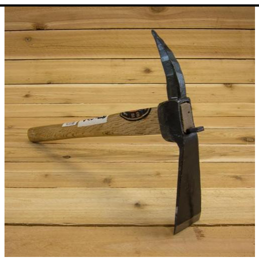	Tool Pickaxe Wooden Metal Digging

Continued on next page

Image Label	Test Image in ThingsEEG	Category-based label
00135_pie	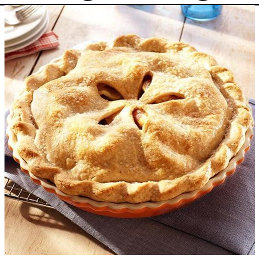	Dessert Pie Baked Crust Golden
00136_pigeon	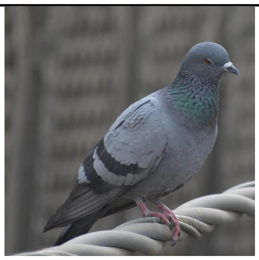	Bird Pigeon Grey Perched Feathers
00137_piglet	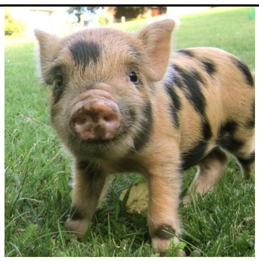	Animal Piglet Spotted Grass Cute
00138_pocket	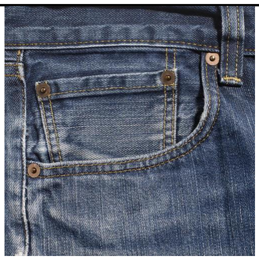	Clothing Jeans Pocket Denim Stitched
00139_pocketknife	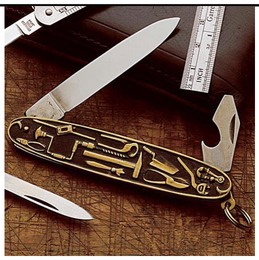	Tool Pocketknife Blade Compact Multi-functional
00140_popcorn	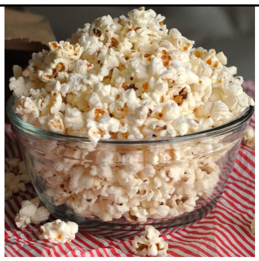	Snack Popcorn Bowl Buttery Crispy

Continued on next page

Image Label	Test Image in ThingsEEG	Category-based label
00141_popsicle	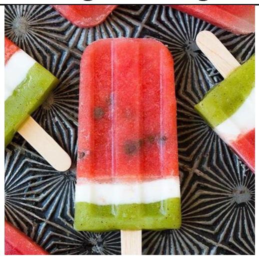	Dessert Popsicle Colorful<br>Frozen Fruit
00142_possum	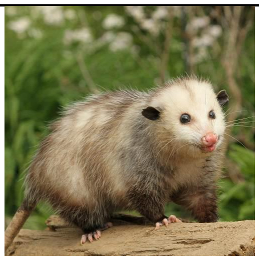	Animal Possum Furry<br>Marsupial Wild
00143_pretzel	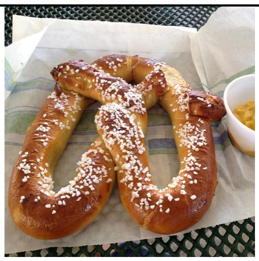	Snack Pretzel Salted<br>Baked Dough
00144_pug	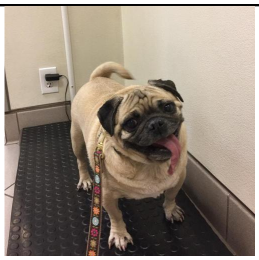	Animal Pug Dog<br>Leash Panting
00145_punch2	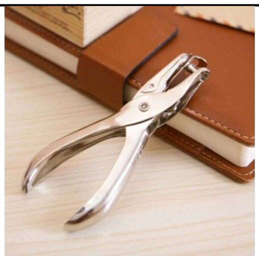	Tool Punch Metal<br>Office Desk
00146_purse	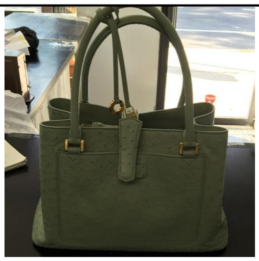	Accessory Purse Leather<br>Green Handles

Continued on next page

Image Label	Test Image in ThingsEEG	Category-based label
00147_radish	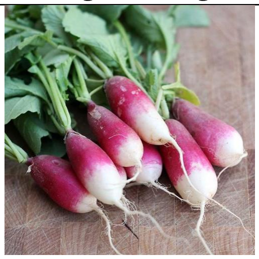	Vegetable Radish Root Fresh Bunch
00148_raspberry	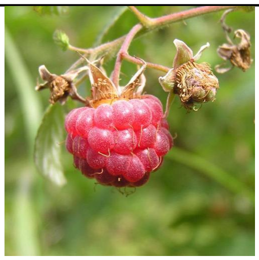	Fruit Raspberry Red Berry Branch
00149_recorder	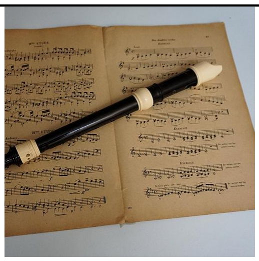	Instrument Recorder Music Notes Sheet
00150_rhinoceros	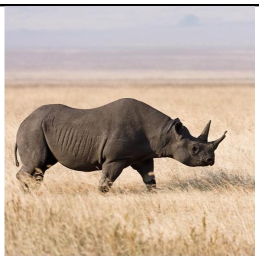	Animal Rhinoceros Horned Savanna Wild
00151_robot	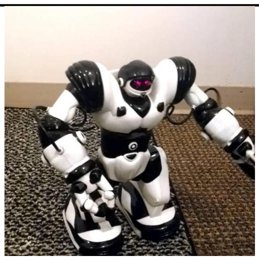	Robot Toy Humanoid Black White
00152_rooster	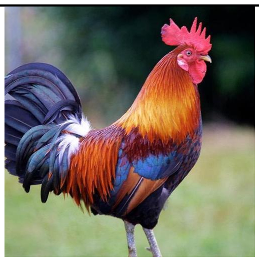	Bird Rooster Feathers Colorful Comb

Continued on next page

Image Label	Test Image in ThingsEEG	Category-based label
00153_rug	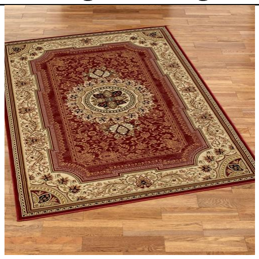	Furniture Rug Patterned Red Ornate
00154_sailboat	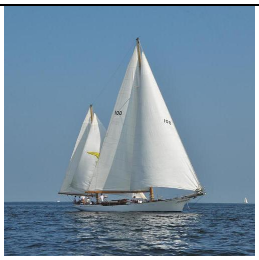	Boat Sailboat Ocean White Wind
00155_sandal	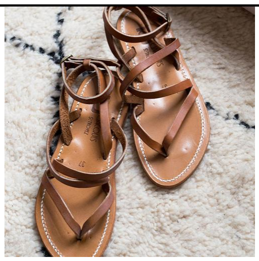	Footwear Sandals Leather Straps Brown
00156_sandpaper	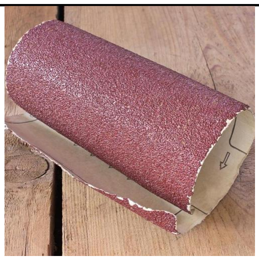	Tool Sandpaper Abrasive Roll Rough
00157_sausage	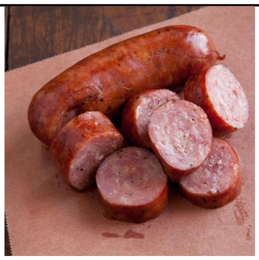	Food Sausage Sliced Smoked Meat
00158_scallion	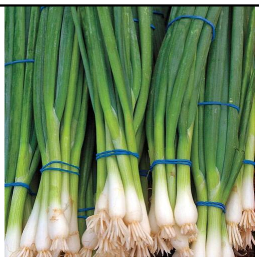	Vegetable Scallion Green Fresh Bundle

Continued on next page

Image Label	Test Image in ThingsEEG	Category-based label
00159_scallop	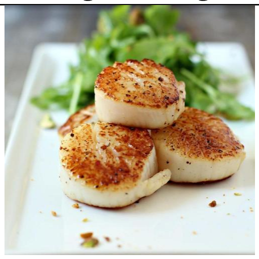	Seafood Scallops Seared Plate Garnish
00160_scooter	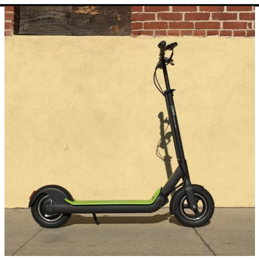	Vehicle Scooter Electric Green Urban
00161_seagull	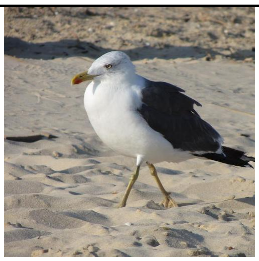	Bird Seagull Beach White Walking
00162_seaweed	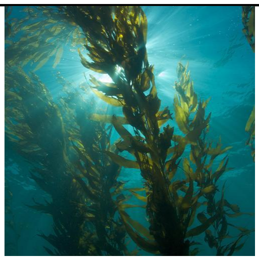	Marine Seaweed Underwater Aquatic Sunlight
00163_seed	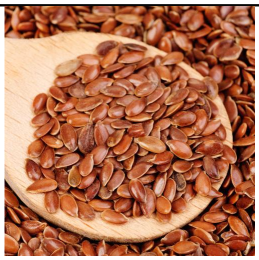	Food Seeds Flax Brown Spoon
00164_skateboard	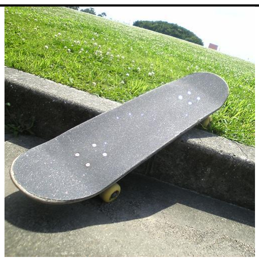	Sport Skateboard Wheels Outdoor Deck

Continued on next page

Image Label	Test Image in ThingsEEG	Category-based label
00165_sled	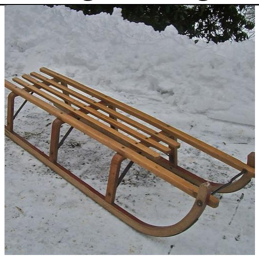	Winter Sled Wooden<br>Snow Sleigh
00166_sleeping_bag	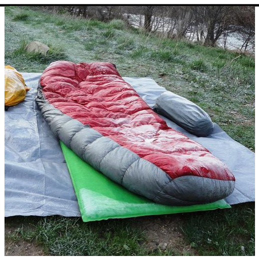	Camping Sleeping Bag<br>Outdoor Frost
00167_slide	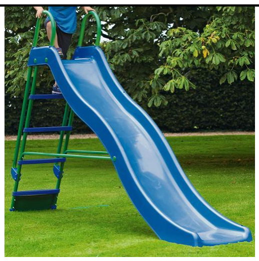	Playground Slide Blue<br>Ladder Outdoor
00168_slingshot	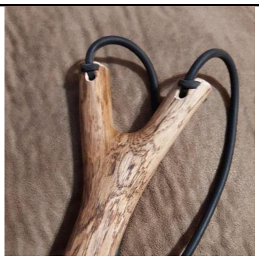	Tool Slingshot Wooden<br>Rubber Y-shaped
00169_snowshoe	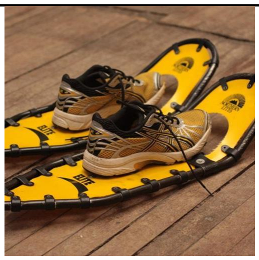	Footwear Snowshoes Yellow<br>Running Winter
00170_spatula	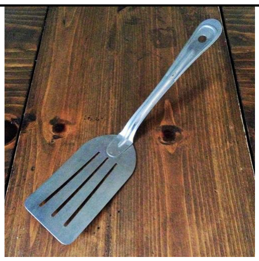	Utensil Spatula Metal<br>Slotted Handle

Continued on next page

Image Label	Test Image in ThingsEEG	Category-based label
00171_spoon	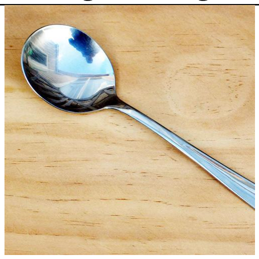	Utensil Spoon Metal Reflection Curved
00172_station_wagon	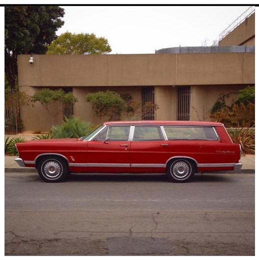	Vehicle Station Wagon Red Classic
00173_stethoscope	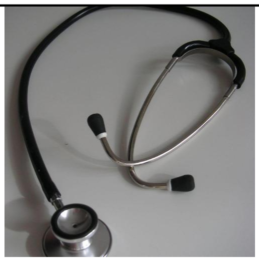	Medical Stethoscope Instrument Black Diagnosis
00174_strawberry	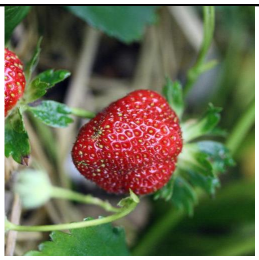	Fruit Strawberry Red Ripe Plant
00175_submarine	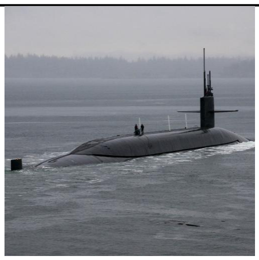	Vessel Submarine Navy Water Stealth
00176_suit	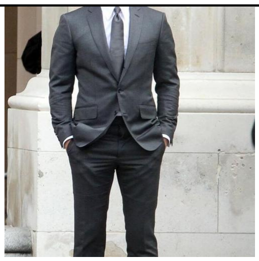	Clothing Suit Formal Business Tailored

Continued on next page

Image Label	Test Image in ThingsEEG	Category-based label
00177_t-shirt	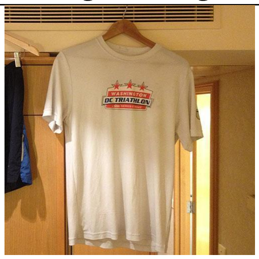	Clothing T-shirt White<br>Event Hanger
00178_table	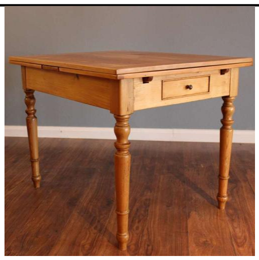	Furniture Table Wooden<br>Square Drawer
00179_taillight	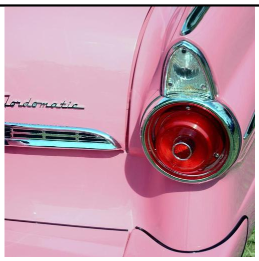	Vehicle Taillight Pink<br>Classic Chrome
00180_tape_recorder	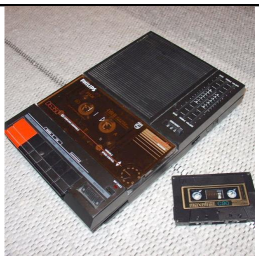	Device Recorder Cassette<br>Vintage Audio
00181_television	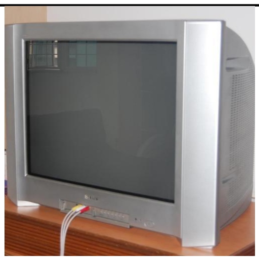	Electronics Television CRT<br>Screen Retro
00182_tiara	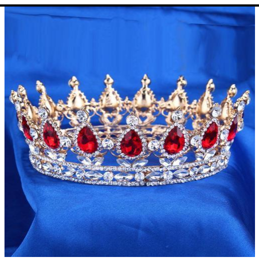	Crown Tiara Gold<br>Jewels Red

Continued on next page

Image Label	Test Image in ThingsEEG	Category-based label
00183_tick	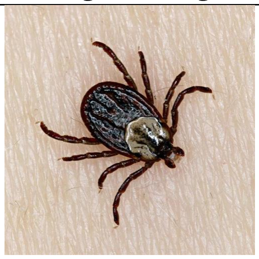	Insect Tick Parasite Skin Tiny
00184_tomato_sauce	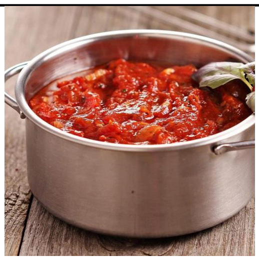	Food Sauce Tomato Pot Red
00185_tongs	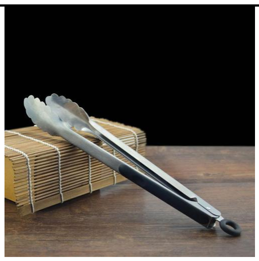	Utensil Tongs Metal Grip Kitchen
00186_tool	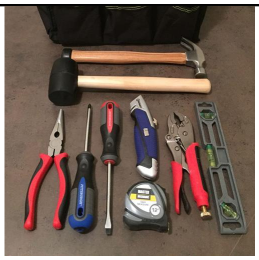	Tools Hammer Pliers Screwdriver Utility
00187_top_hat	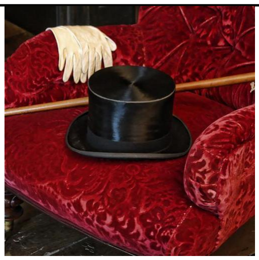	Accessory Top-hat Cane Gloves Velvet
00188_treadmill	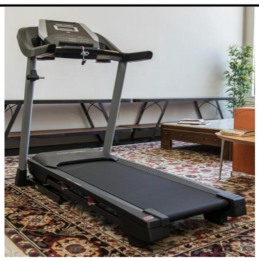	Exercise Treadmill Machine Indoor Fitness

Continued on next page

Image Label	Test Image in ThingsEEG	Category-based label
00189_tube_top	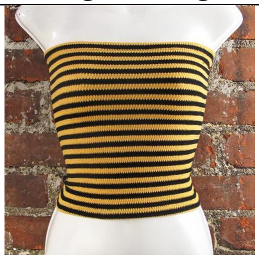	Clothing Top Striped<br>Yellow Knitted
00190_turkey	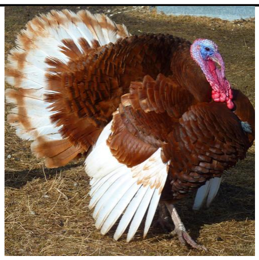	Bird Turkey Feathers<br>Fanned Brown
00191_unicycle	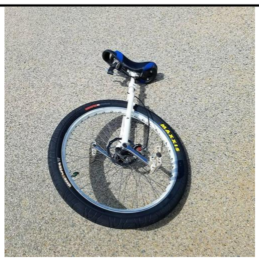	Vehicle Unicycle Wheel<br>Tire Seat
00192_vise	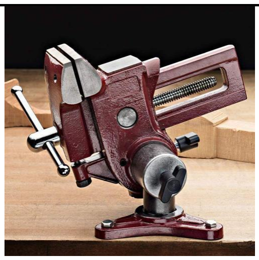	Tool Vise Metal<br>Clamp Adjustable
00193_volleyball	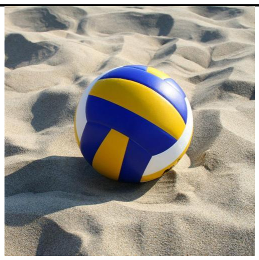	Sport Volleyball Beach<br>Ball Sand
00194_wallpaper	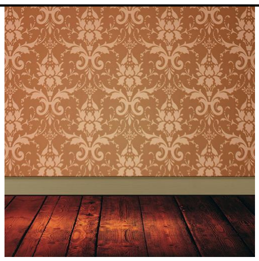	Interior Wallpaper Pattern<br>Vintage Wood

Continued on next page

Image Label	Test Image in ThingsEEG	Category-based label
00195_walnut	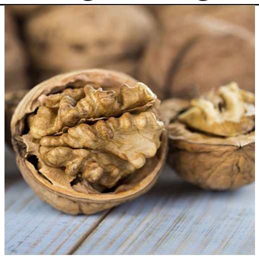	Food Walnut Nut<br>Shell Brown
00196_wheat	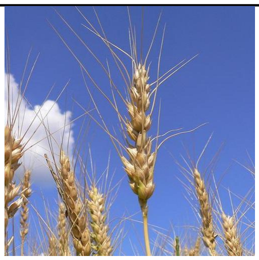	Crop Wheat Grain<br>Field Stalk
00197_wheelchair	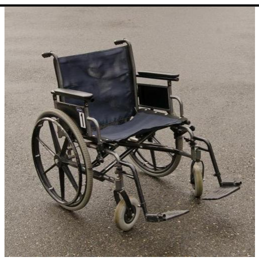	Mobility Wheelchair Manual<br>Wheels Seat
00198_windshield	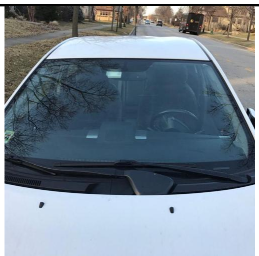	Vehicle Windshield Glass<br>Car Street
00199_wine	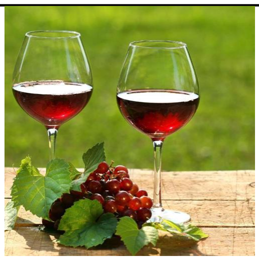	Beverage Wine Glass<br>Grapes Red
00200_wok	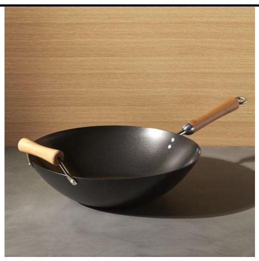	Cookware Wok Pan<br>Handles Black
